# Supplementary material for: Potential of Ramalin and Its Derivatives for the Treatment of Alzheimer’s Disease
Source: Molecules. 2021 Oct 26;26(21):6445. doi: 10.3390/molecules26216445 (PMC8588271; doi:10.3390/molecules26216445)
Supplement: Supplementary file 1 [file molecules-26-06445-s001.zip › molecules-1398968-supplementary.pdf]

Supplymentary Information

Potential of Ramalin and Its Derivatives for the Treatment of Alzheimer's Disease

Tai Kyoung Kim [1,†], Ju-Mi Hong [1,†], Kyung Hee Kim [1,3], Se Jong Han [1], Il-Chan Kim [1], Hyuncheol Oh [2] and Joung Han Yim [1,*]

[1] Division of Polar Life Sciences, Korea Polar Research Institute, Incheon 21990, Korea; tkkim@kopri.re.kr (T.K.K.); wnal5555@kopri.re.kr (J.-M.H.); kh313@kopri.re.kr (K.H.K.); hansj@kopri.re.kr (S.J.H.); ickim@kopri.re.kr (I.-C.K.);
[2] College of Pharmacy, Wonkwang University, Iksan 54538, Korea; hoh@wcu.ac.kr
[3] Department of chemistry, Hanseo University, Seosan 31962, Korea
* Correspondence: jhyim@kopri.re.kr; Tel.: +82-32-760-5540; Fax: +82-32-760-5509
† These authors contributed equally to this study.

Context ...... page

N⁵-(o-tolylamino)-L-glutamine (**RA-2Me**)

Figure S1. ^{1}H NMR spectrum of **RA-2Me** 4

Figure S2. ^{13}C NMR spectrum of **RA-2Me** 4

Figure S3. DEPT spectrum of **RA-2Me** 5

Figure S4. COSY spectrum of **RA-2Me** 5

Figure S5. HRMS spectrum of **RA-2Me** 6

Figure S6. IR spectrum of **RA-2Me** 6

N⁵-(m-tolylamino)-L-glutamine (**RA-3Me**)

Figure S7. ^{1}H NMR spectrum of **RA-3Me** 7

Figure S8. ^{13}C NMR spectrum of **RA-3Me** 7

Figure S9. DEPT spectrum of **RA-3Me** 8

Figure S10. HRMS spectrum of **RA-3Me** 8

Figure S11. IR spectrum of **RA-3Me** 9

N⁵-(p-tolylamino)-L-glutamine (**RA-4Me**)

Figure S12. ^{1}H NMR spectrum of **RA-4Me** 9

Figure S13. ^{13}C NMR spectrum of **RA-4Me** 10

Figure S14. DEPT spectrum of **RA-4Me** 10

Figure S15. HRMS spectrum of **RA-4Me** 11

Figure S16. IR spectrum of **RA-4Me** 11

N⁵-((2,5-dimethylphenyl)amino)-L-glutamine (**RA-25Me**)

Figure S17. ^{1}H NMR spectrum of **RA-25Me** 12

Figure S18. ^{13}C NMR spectrum of **RA-25Me** 12

Figure S19. DEPT spectrum of **RA-25Me** 13

Figure S20. HRMS spectrum of **RA-25Me** 13

Figure S21. IR spectrum of **RA-25Me** 14

N⁵-((3,4-dimethylphenyl)amino)-L-glutamine (**RA-34Me**)

Figure S22. ^{1}H NMR spectrum of **RA-34Me** 14

Figure S23. ^{13}C NMR spectrum of **RA-34Me** 15

Figure S24. DEPT spectrum of **RA-34Me** 15

Figure S25. COSY spectrum of **RA-34Me** 16

Figure S26. HSQC spectrum of **RA-34Me** 16

Figure S27. HRMS spectrum of **RA-34Me** 17

Figure S28. IR spectrum of **RA-34Me** 17

N^5-((2-fluorophenyl)amino)-L-glutamine **(RA-2F)**

Table S1. Assignment of NMR data of **RA-2F** 18

Figure S29. ^{1}H NMR spectrum of **RA-2F** 18

Figure S30. ^{13}C NMR spectrum of **RA-2F** 19

Figure S31. DEPT spectrum of **RA-2F** 19

Figure S32. HSQC spectrum of **RA-2F** 20

Figure S33. HRMS spectrum of **RA-2F** 20

Figure S34. IR spectrum of **RA-2F** 21

N^5-((4-fluorophenyl)amino)-L-glutamine **(RA-4F)**

Table S2. Assignment of NMR data of **RA-4F** 21

Figure S35. ^{1}H NMR spectrum of **RA-4F** 22

Figure S36. ^{13}C NMR spectrum of **RA-4F** 22

Figure S37. DEPT spectrum of **RA-4F** 23

Figure S38. HRMS spectrum of **RA-4F** 23

Figure S39. IR spectrum of **RA-4F** 24

N^5-((2,4-difluorophenyl)amino)-L-glutamine **(RA-24F)**

Table S3. Assignment of NMR data of **RA-24F** 24

Figure S40. ^{1}H NMR spectrum of **RA-24F** 25

Figure S41. ^{13}C NMR spectrum of **RA-24F** 25

Figure S42. DEPT spectrum of **RA-24F** 26

Figure S43. HSQC spectrum of **RA-24F** 26

Figure S44. HRMS spectrum of **RA-24F** 27

Figure S45. IR spectrum of **RA-24F** 27

N^5-((perfluorophenyl)amino)-L-glutamine **(RA-PF)**

Table S4. Assignment of NMR data of **RA-PF** 28

Figure S46. ^{1}H NMR spectrum of **RA-PF** 28

Figure S47. ^{13}C NMR spectrum of **RA-PF** 29

Figure S48. DEPT spectrum of **RA-PF** 29

Figure S49. HRMS spectrum of **RA-PF** 30

Figure S50. IR spectrum of **RA-PF** 30

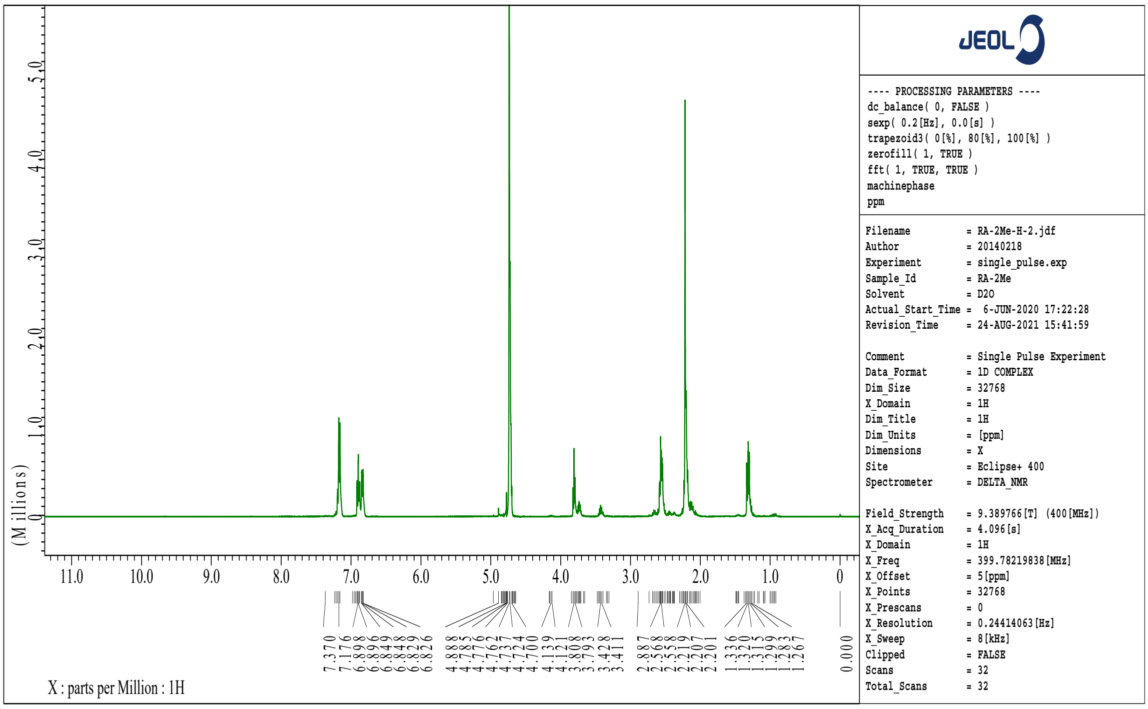

Figure S1: ^{1}H NMR (400 MHz) spectrum of **RA-2Me**

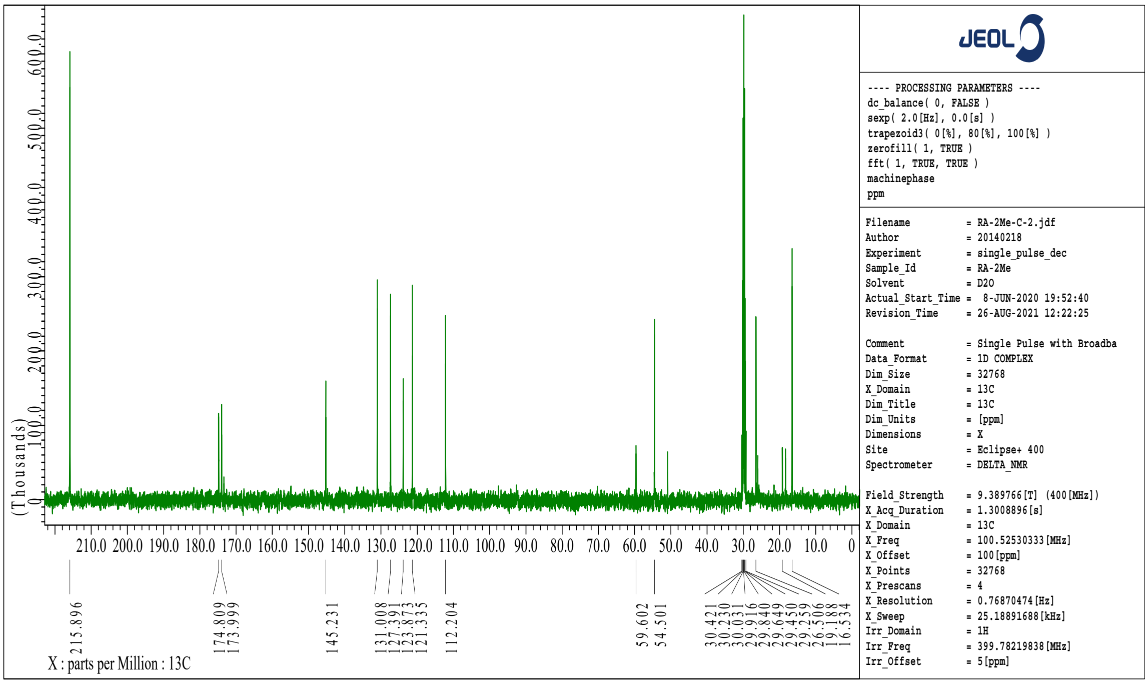

Figure S2: ^{13}C NMR (100 MHz) spectrum of **RA-2Me**

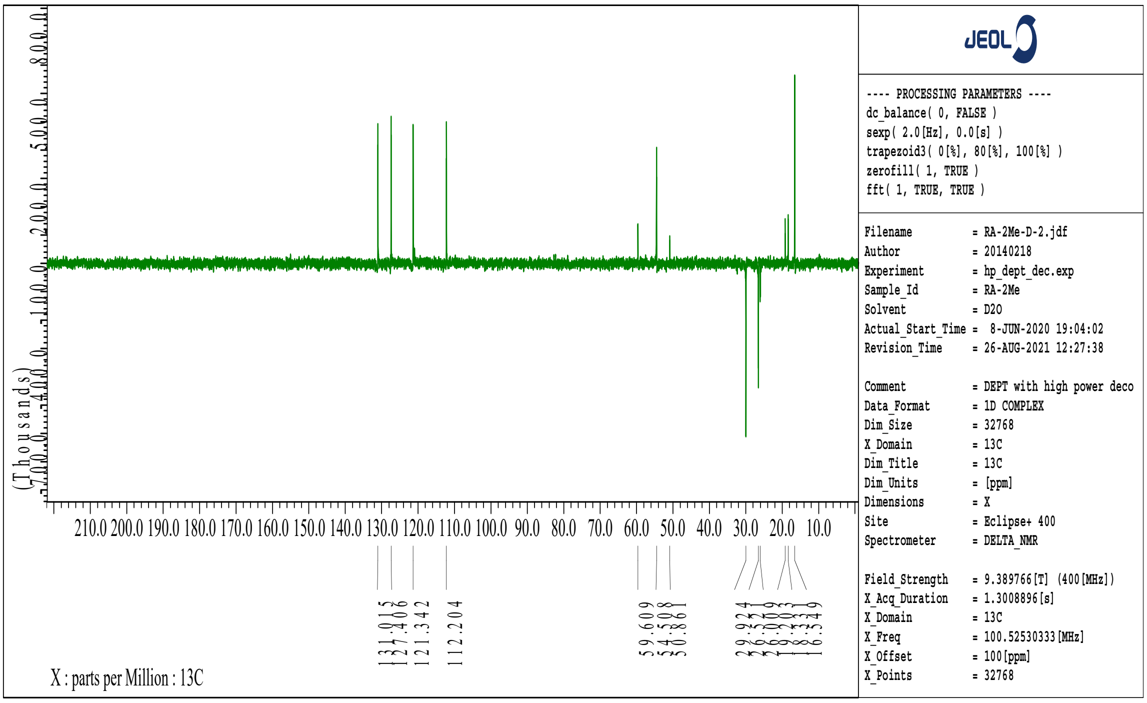

Figure S3: DEPT spectrum of **RA-2Me**

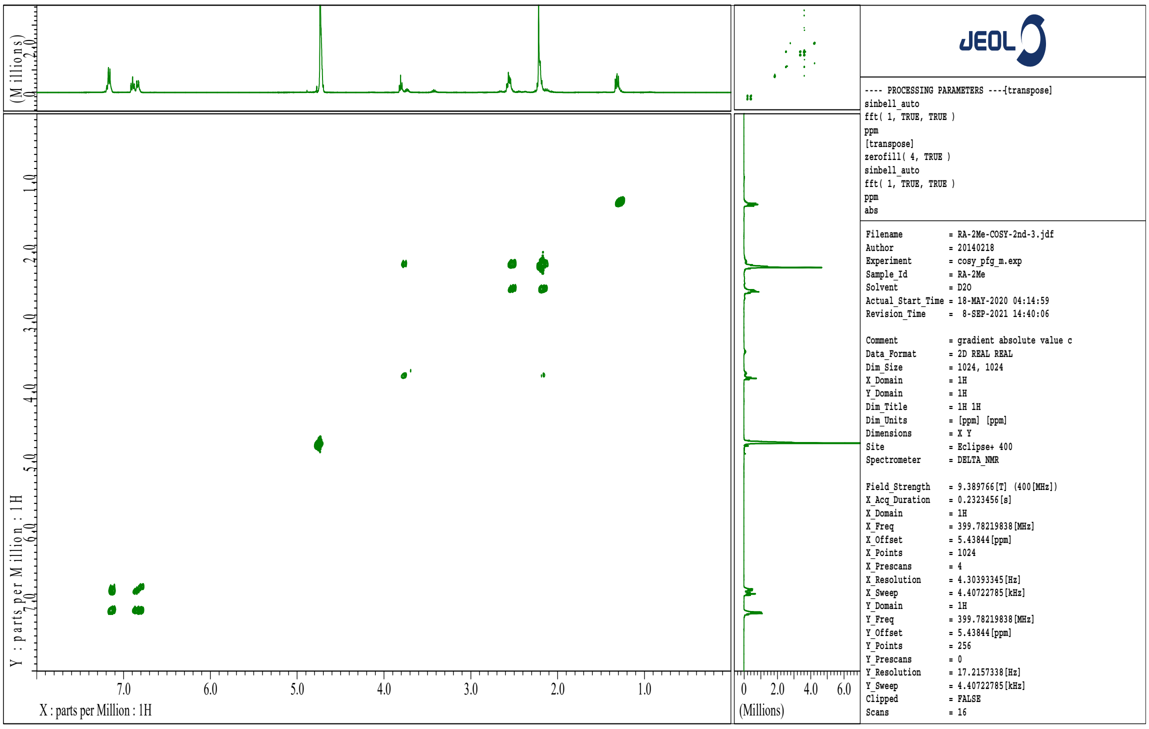

Figure S4: COSY spectrum of **RA-2Me**

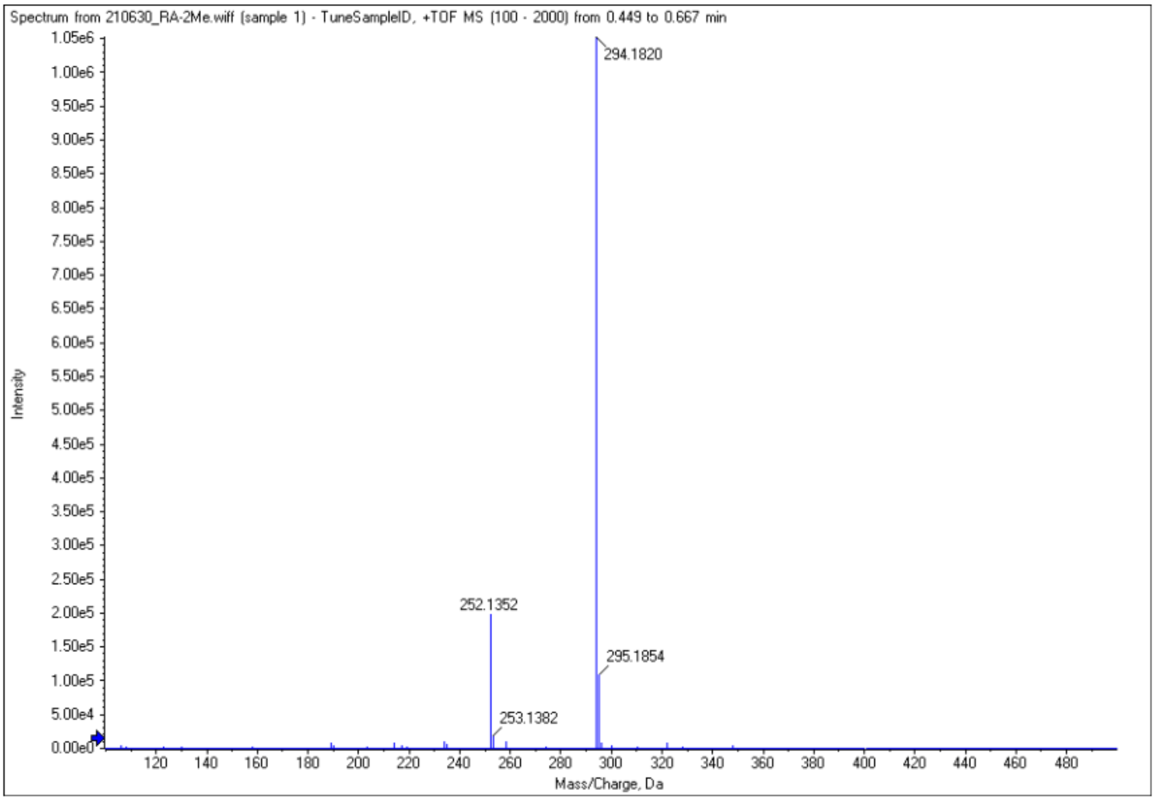

Figure S5: HRMS spectrum of **RA-2Me**

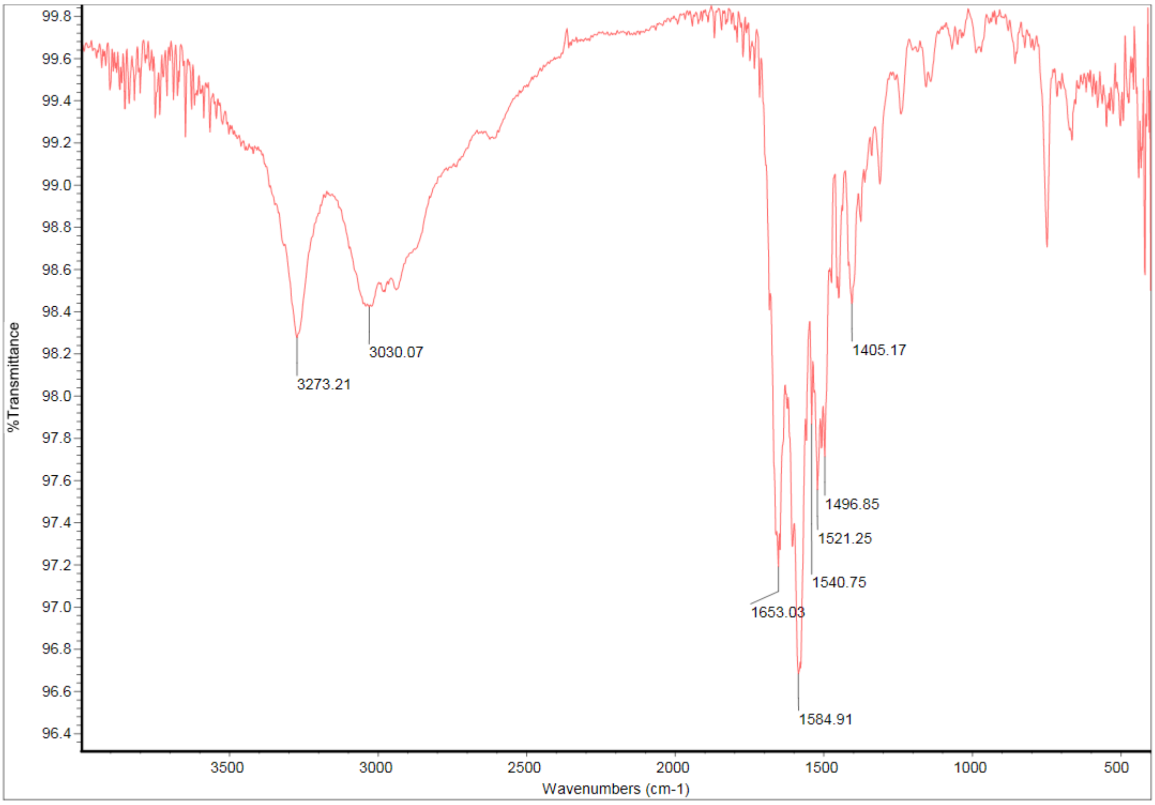

Figure S6: IR spectrum of **RA-2Me**

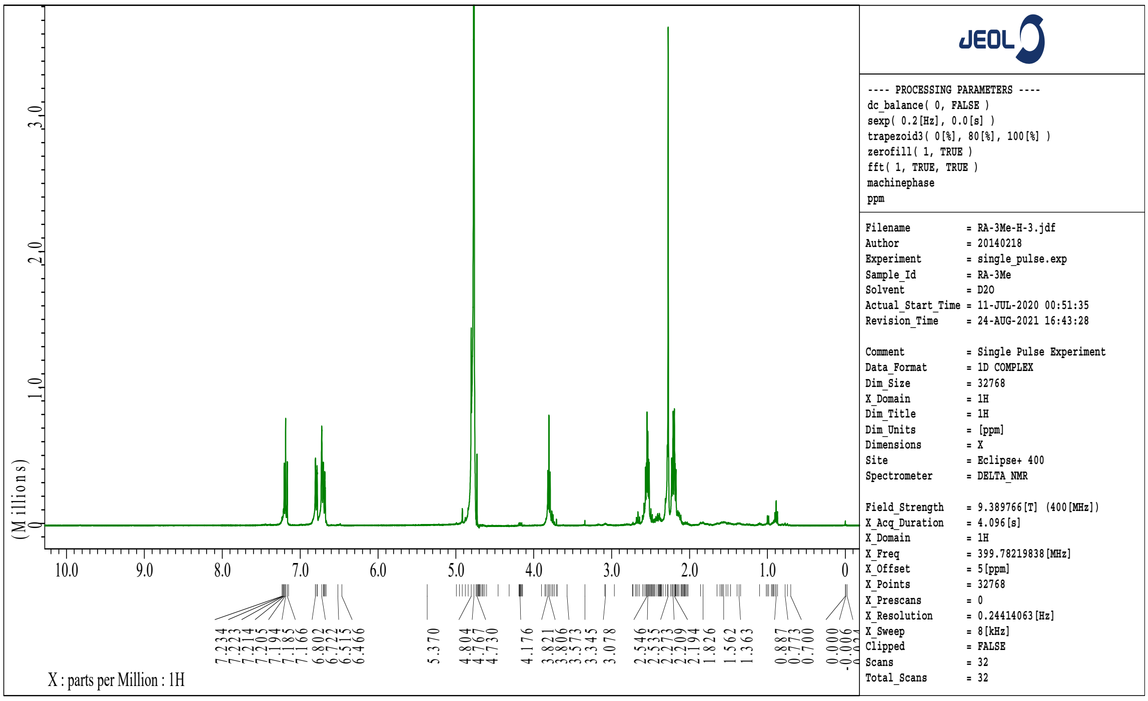

Figure S7: ^{1}H NMR (400 MHz) spectrum of **RA-3Me**

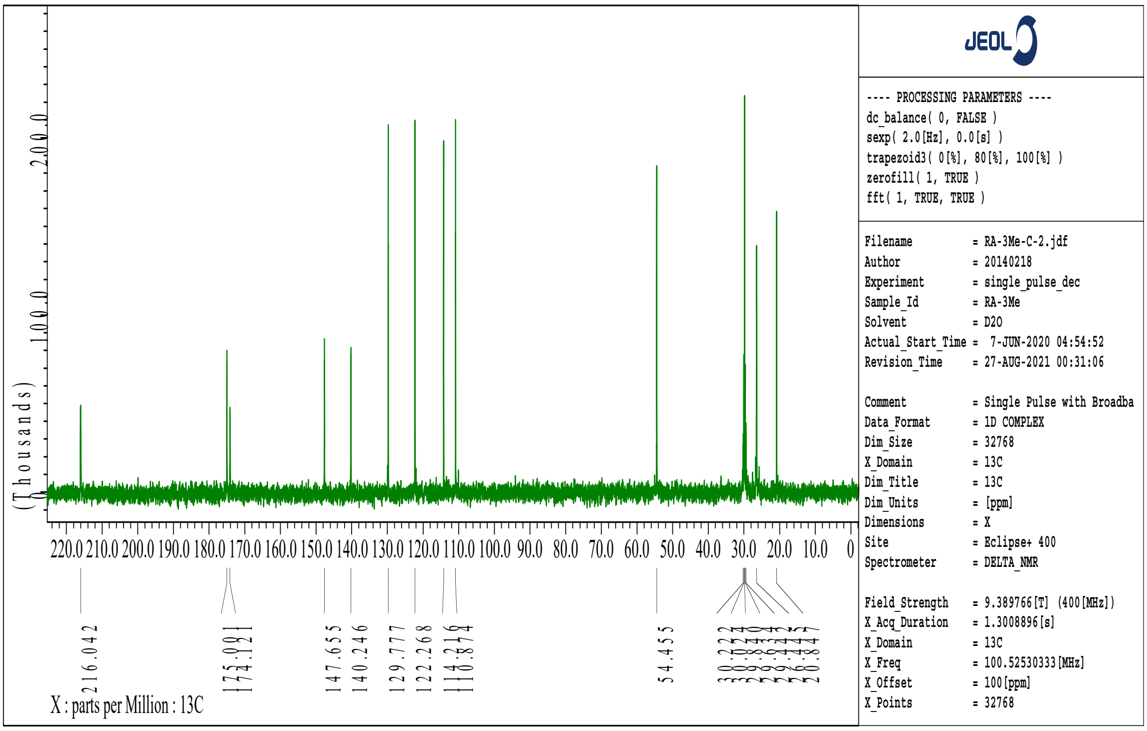

Figure S8: ^{13}C NMR (100 MHz) spectrum of **RA-3Me**

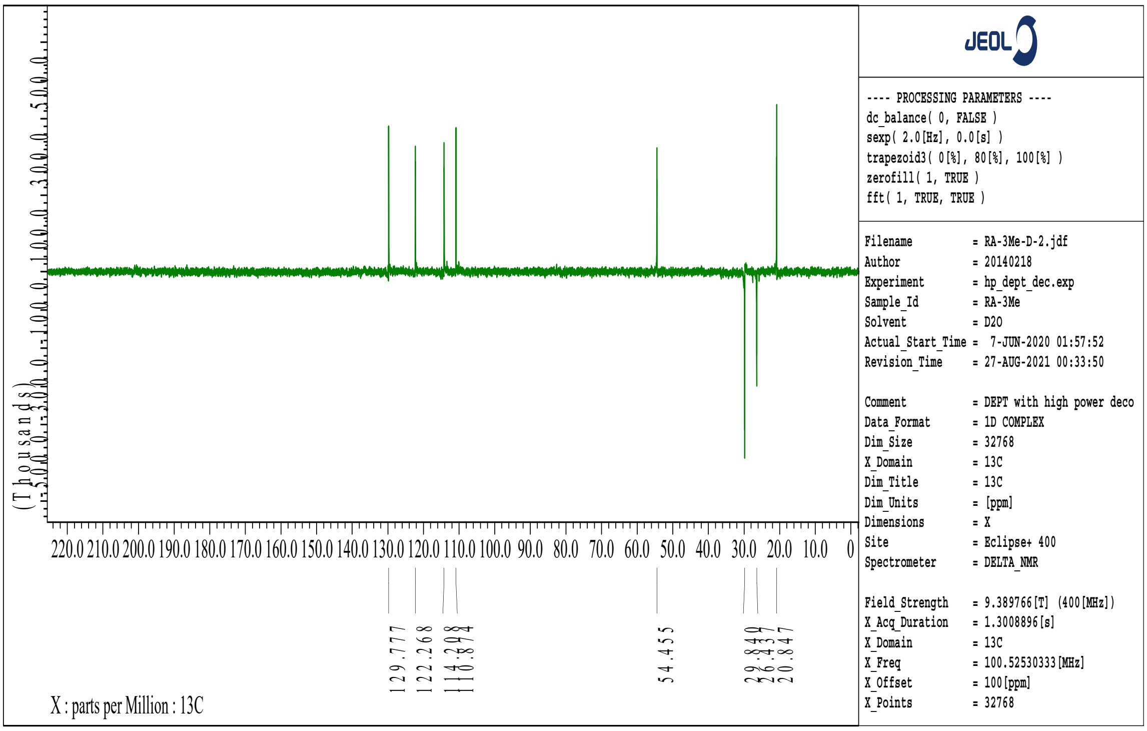

Figure S9: DEPT spectrum of **RA-3Me**

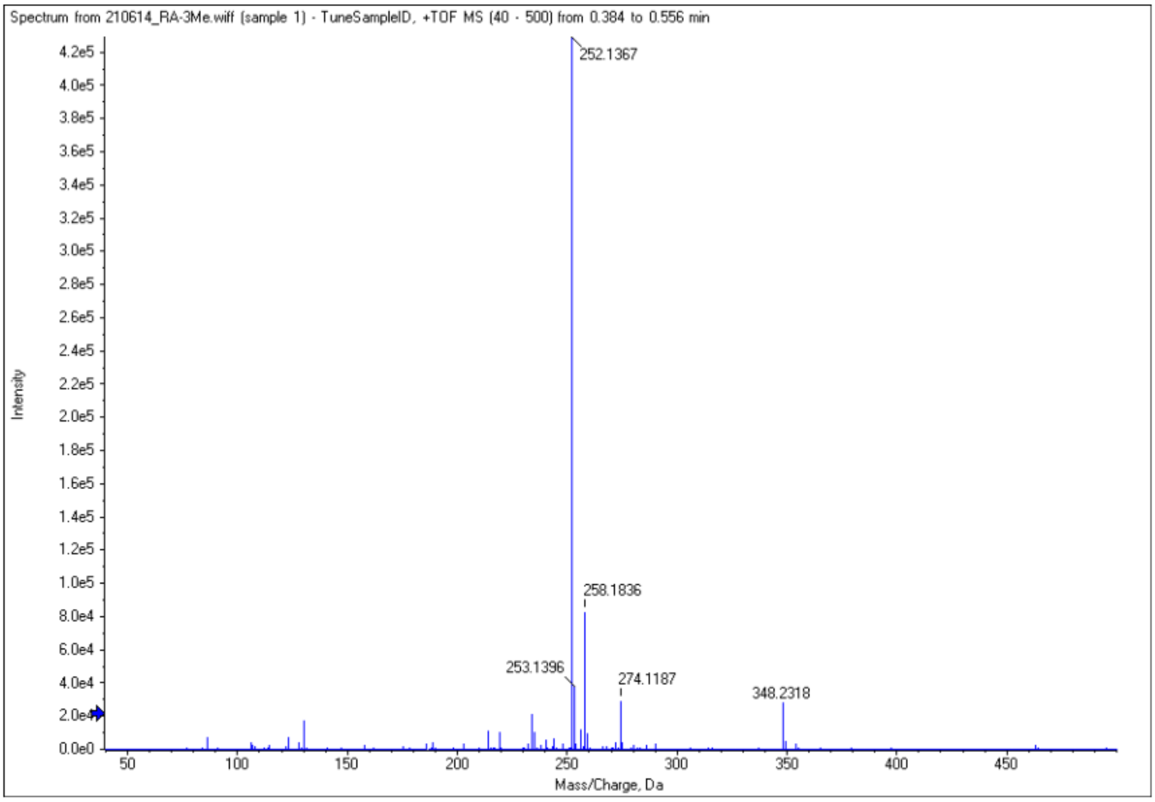

Figure S10: HRMS spectrum of **RA-3Me**

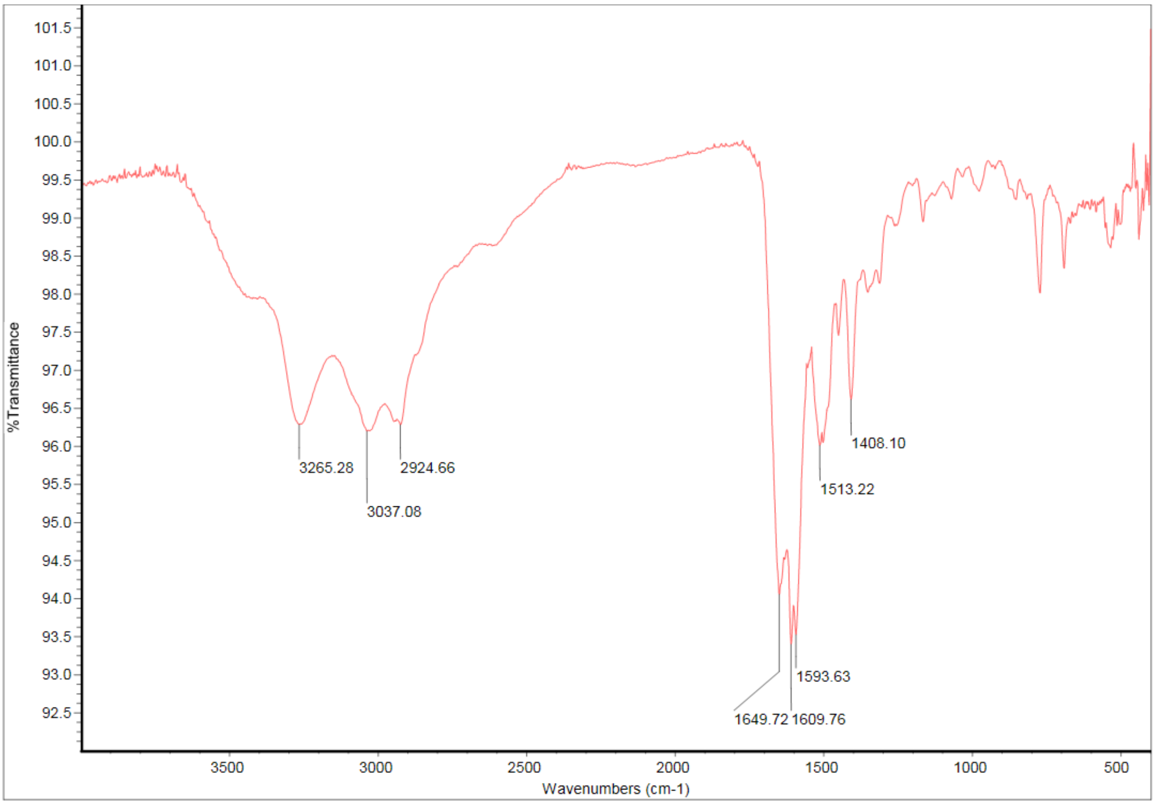

Figure S11: IR spectrum of **RA-3Me**

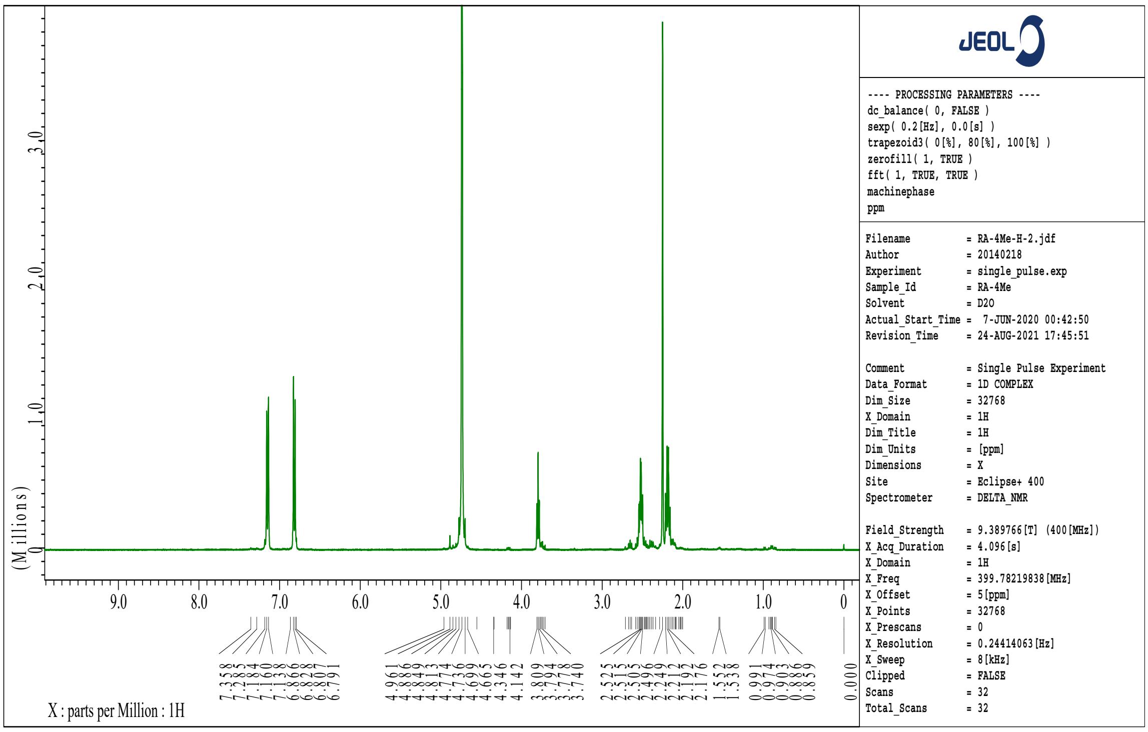

Figure S12: ^{1}H NMR (400 MHz) spectrum of **RA-4Me**

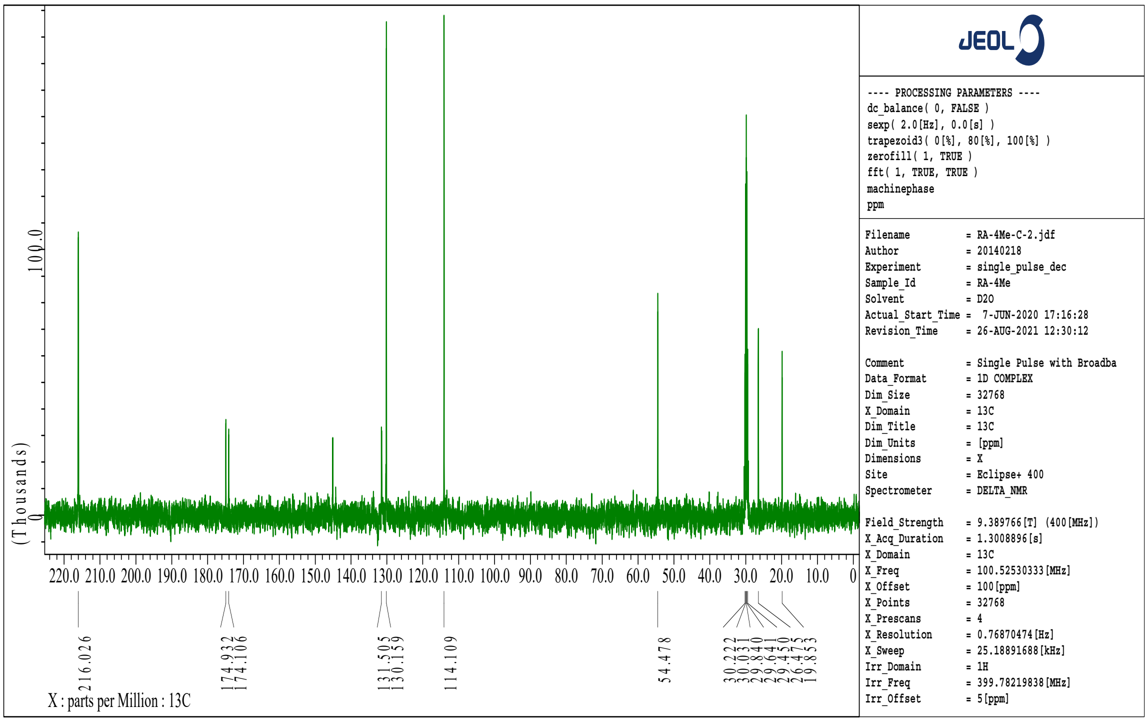

Figure S13: ^{13}C NMR (100 MHz) spectrum of **RA-4Me**

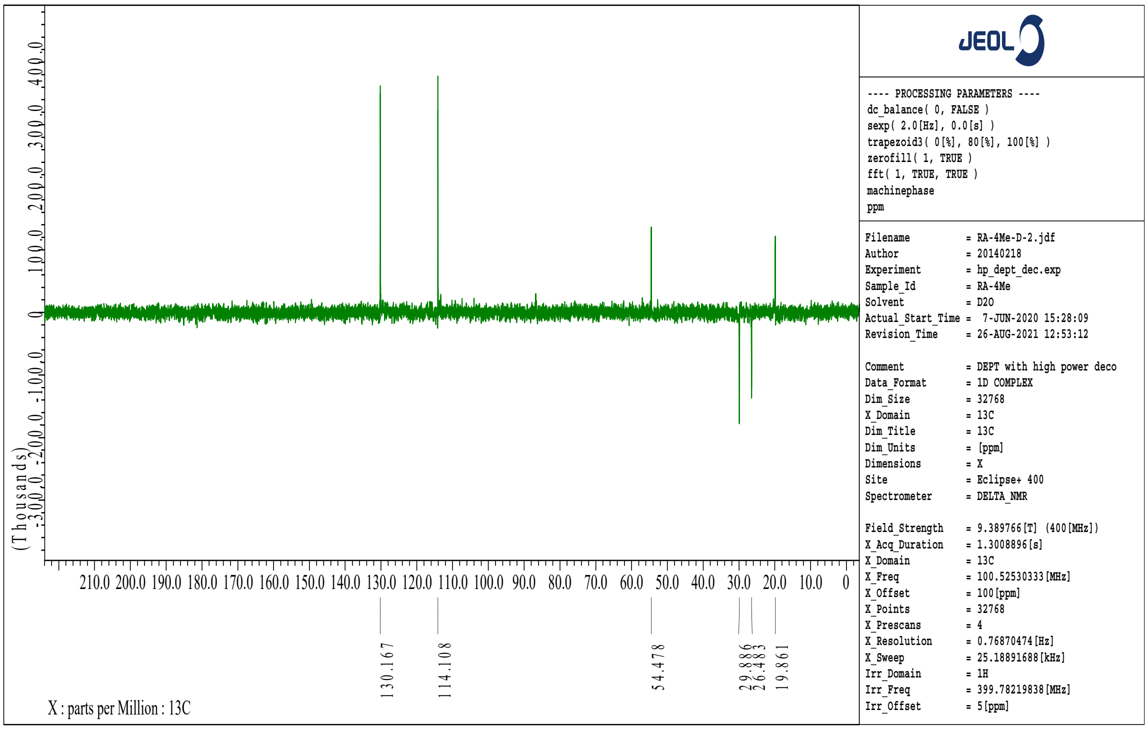

Figure S14: DEPT spectrum of **RA-4Me**

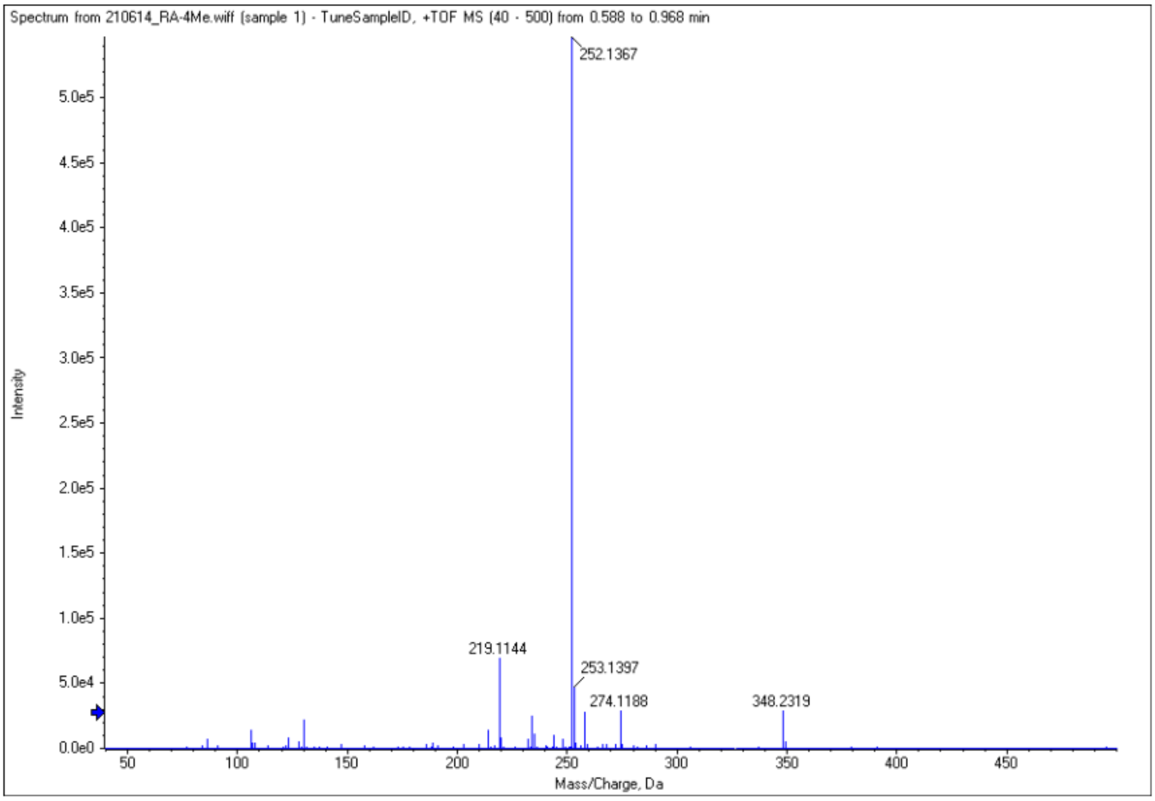

Figure S15: HRMS spectrum of **RA-4Me**

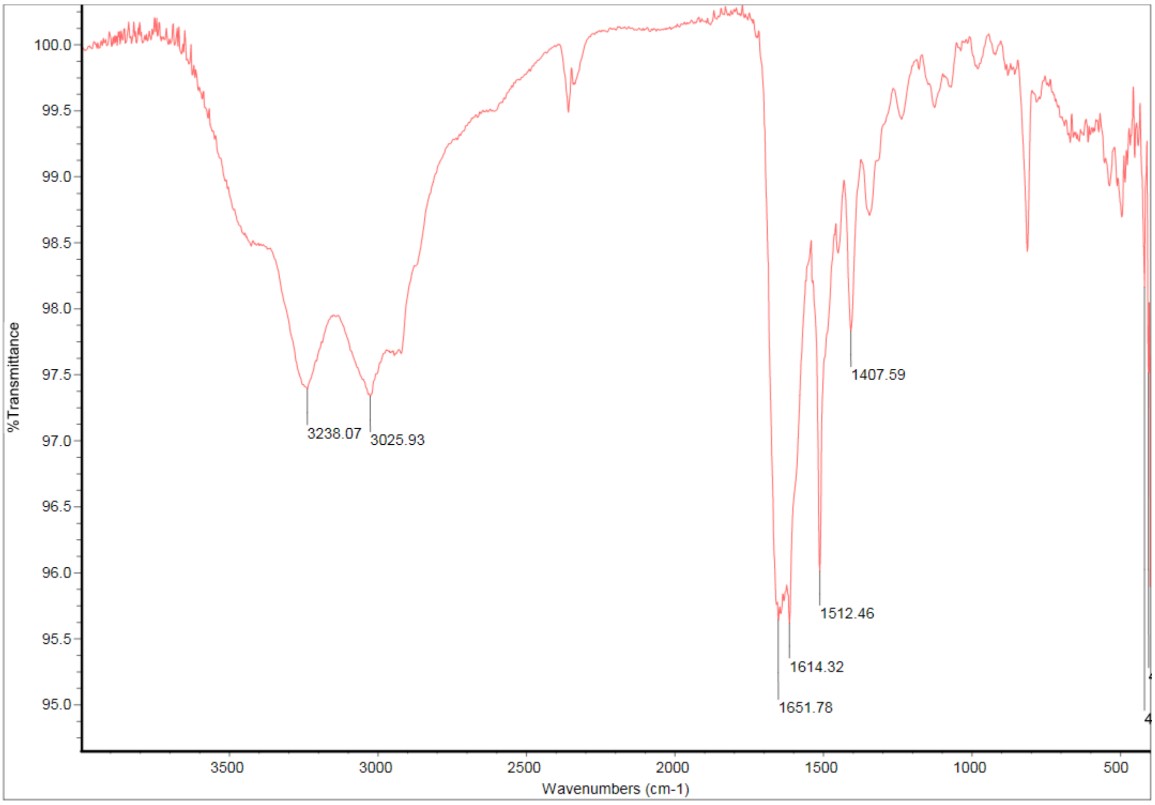

Figure S16: IR spectrum of **RA-4Me**

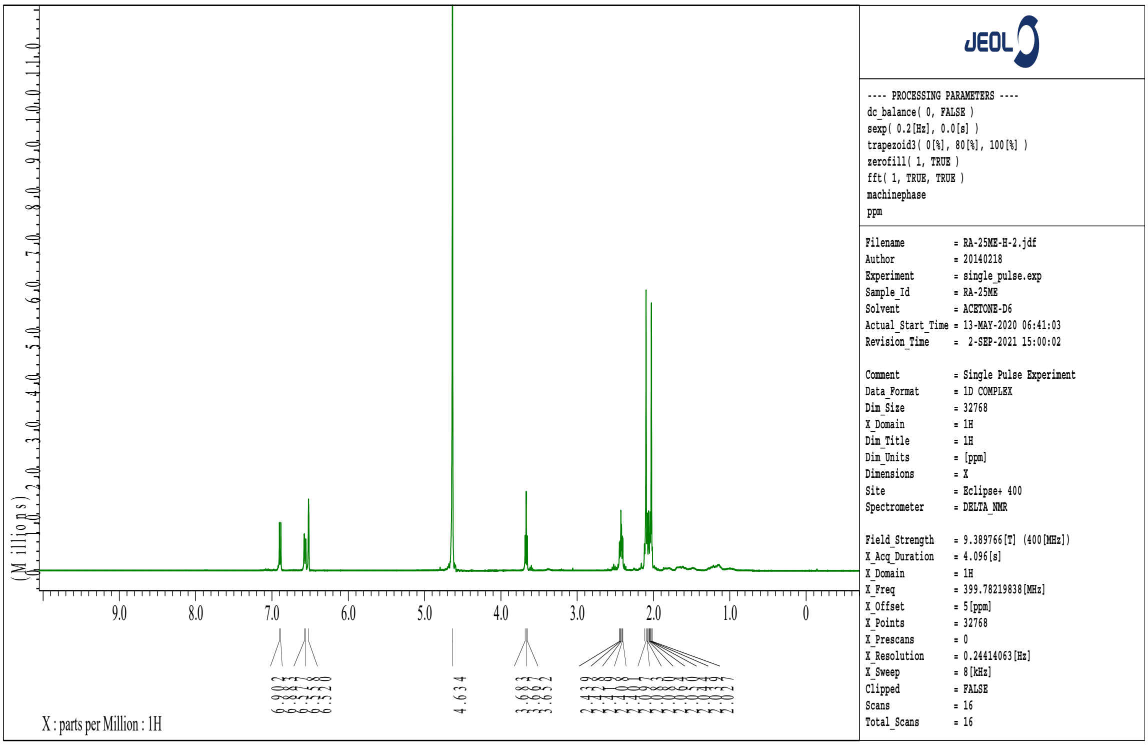

Figure S17: ^{1}H NMR (400 MHz) spectrum of **RA-25Me**

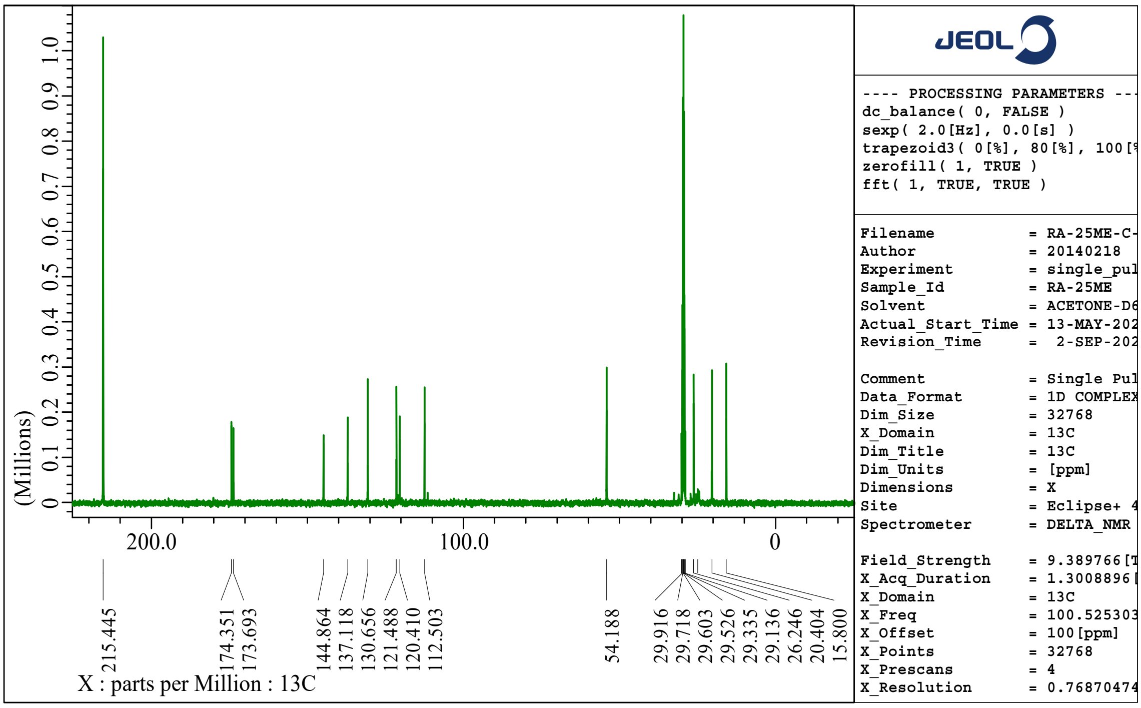

Figure S18: ^{13}C NMR (100 MHz) spectrum of **RA-25Me**

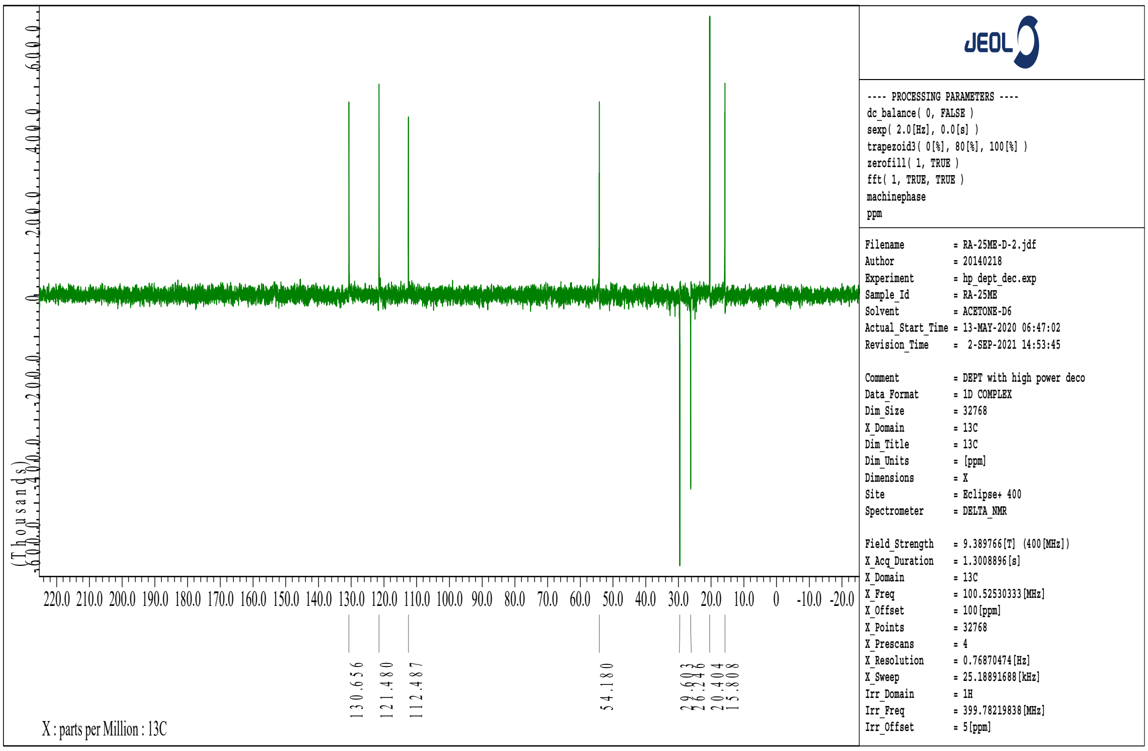

Figure S19: DEPT spectrum of **RA-25Me**

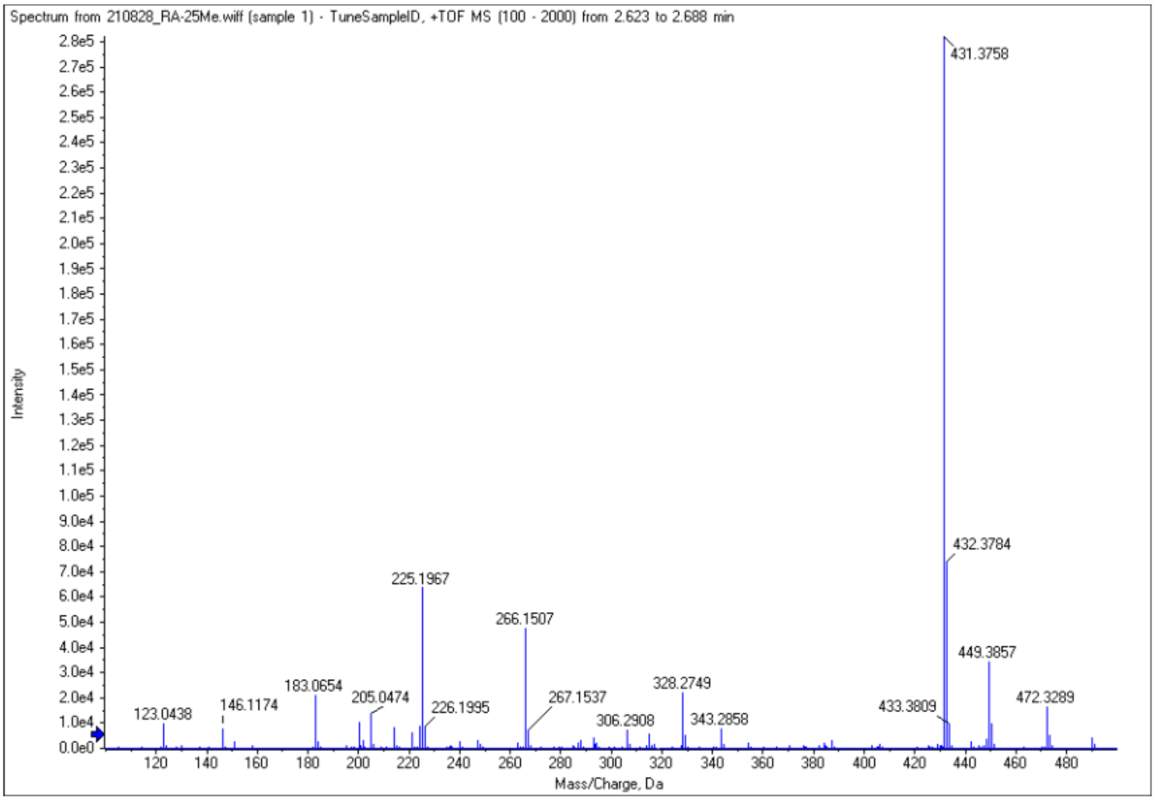

Figure S20: HRMS spectrum of **RA-25Me**

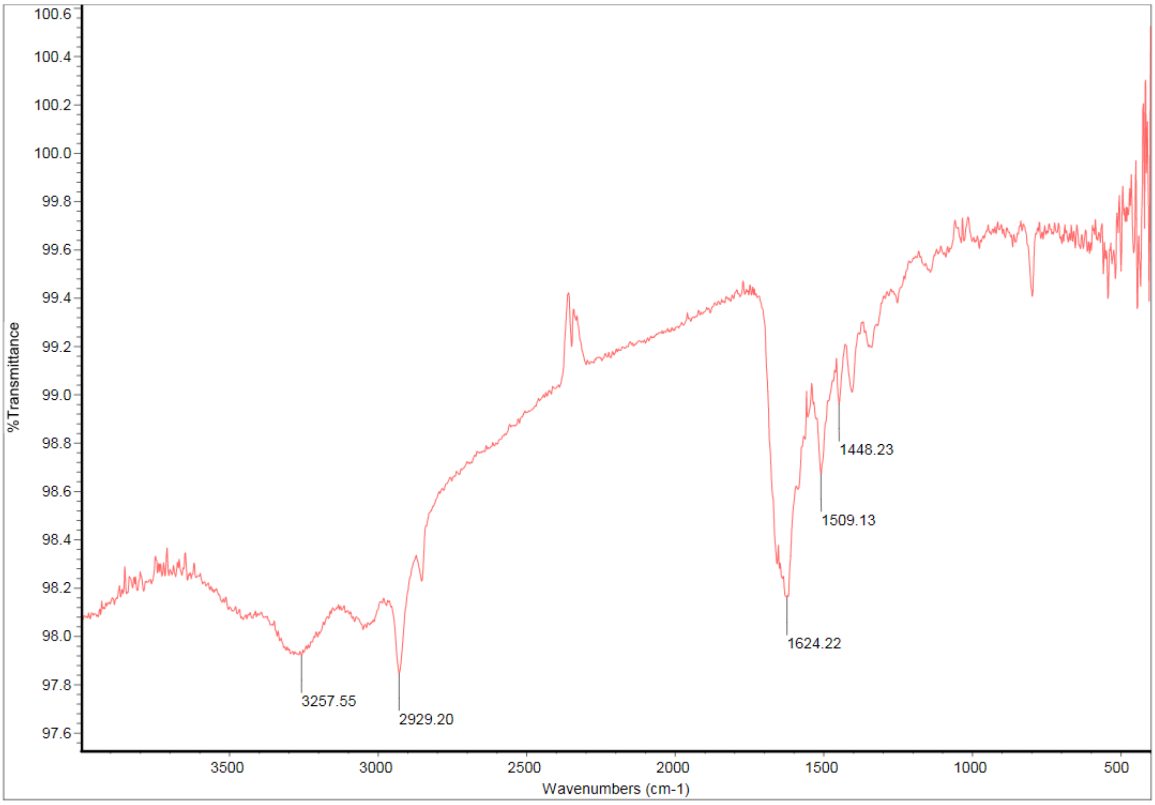

Figure S21: IR spectrum of **RA-25Me**

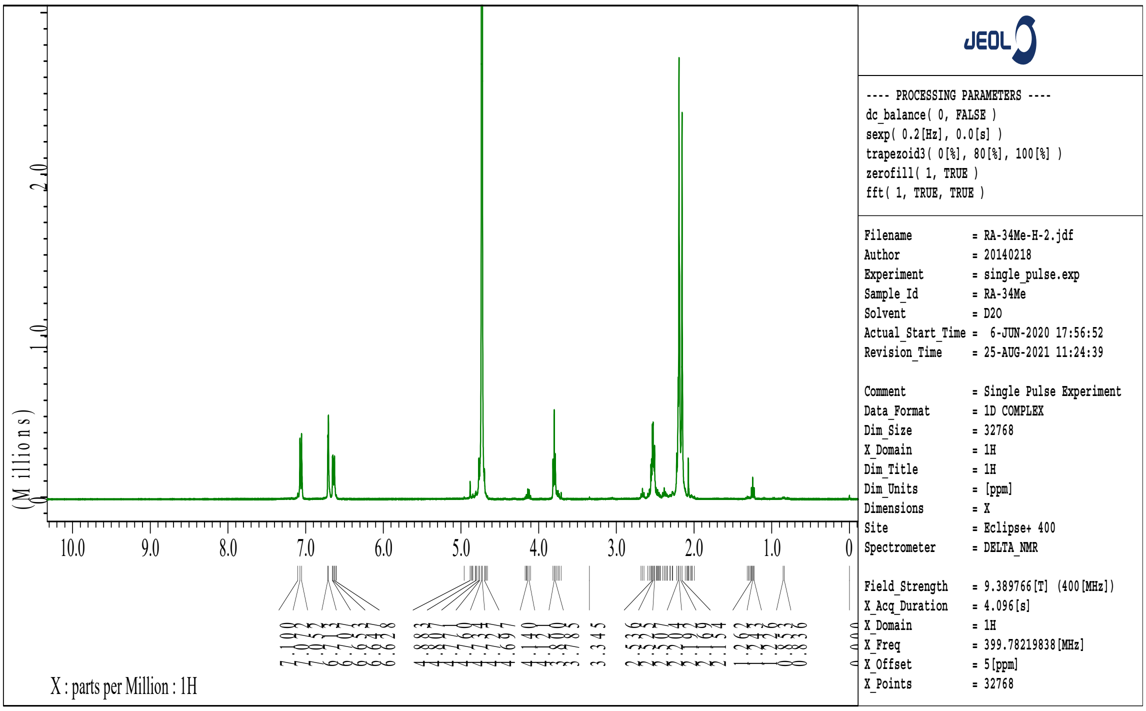

Figure S22: ^{1}H NMR (400 MHz) spectrum of **RA-34Me**

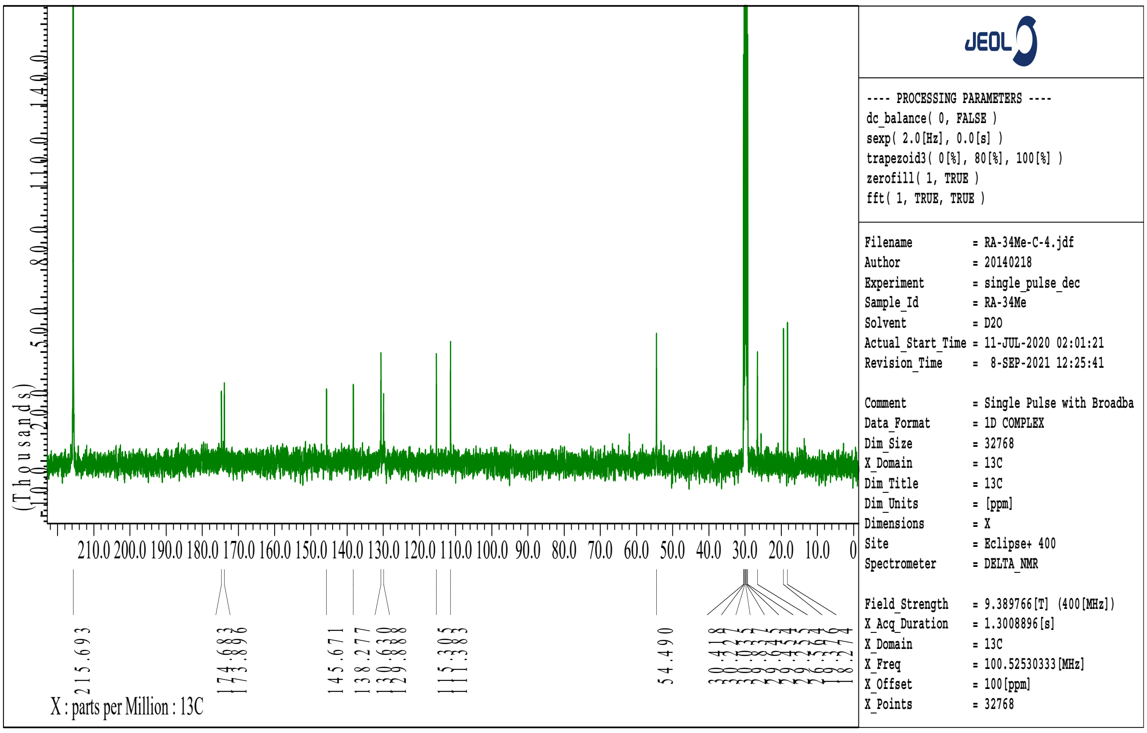

Figure S23: ^{13}C NMR (100 MHz) spectrum of **RA-34Me**

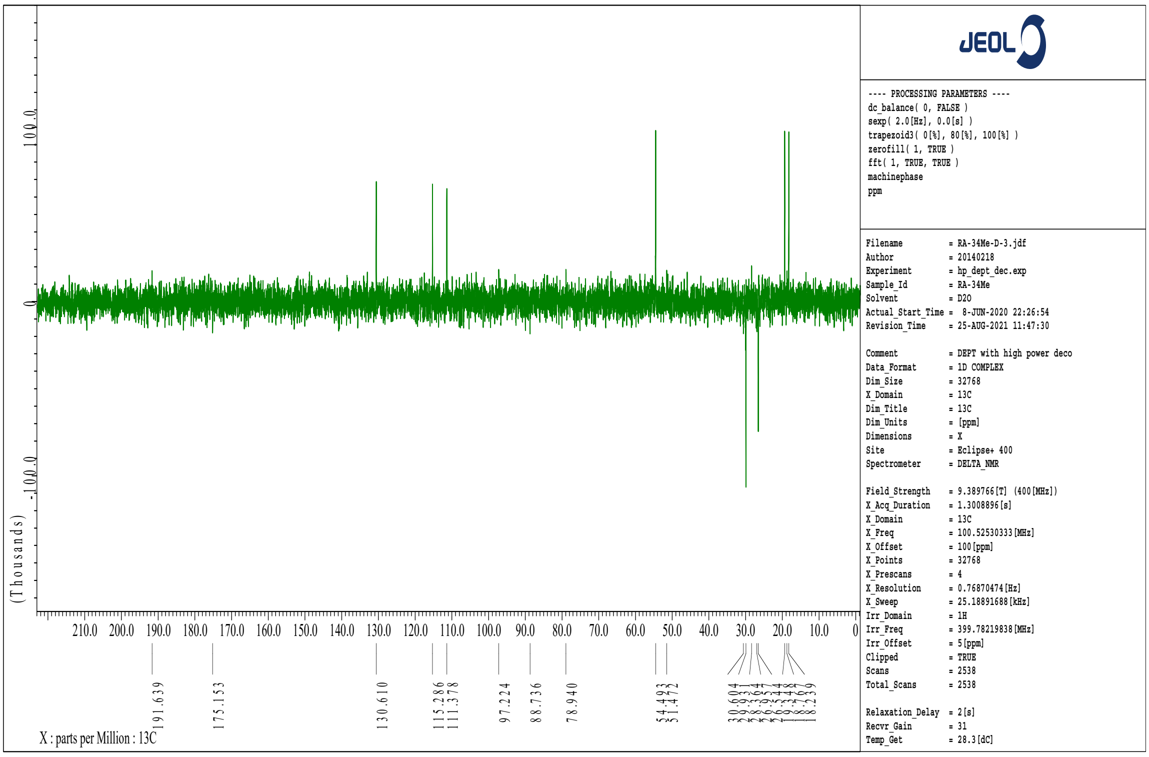

Figure S24: DEPT spectrum of **RA-34Me**

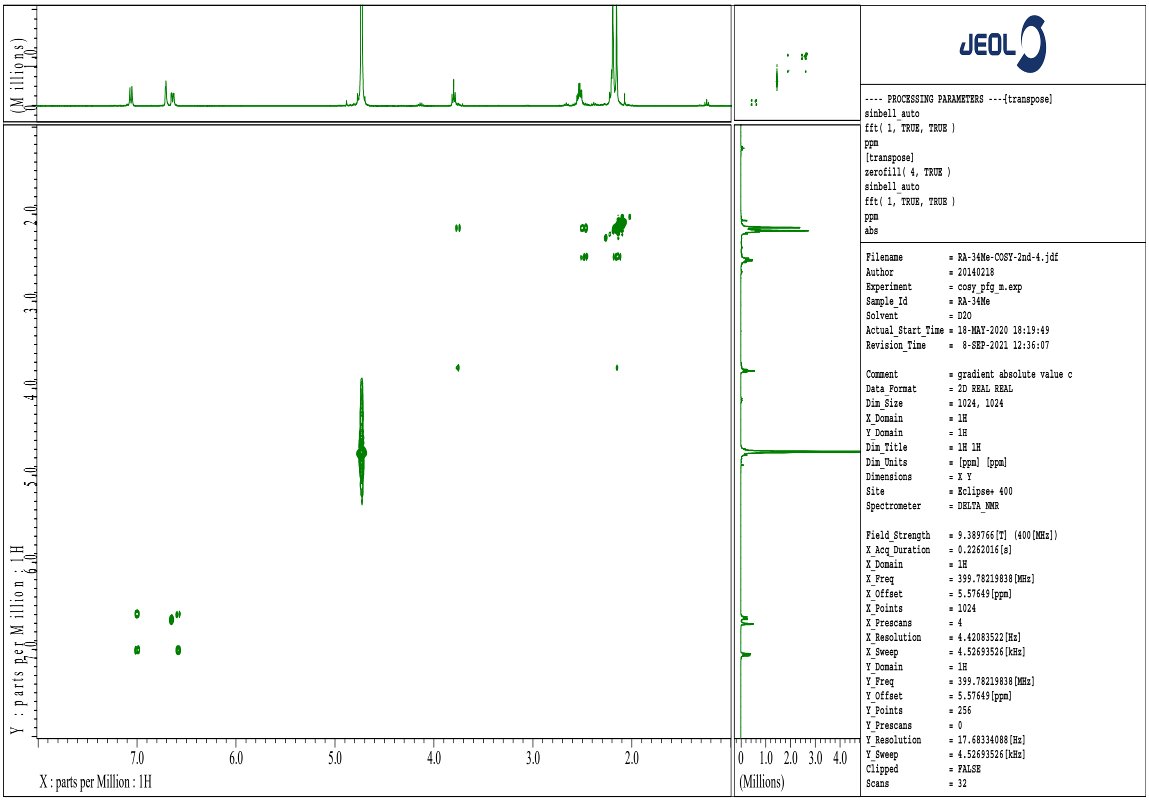

Figure S25: COSY spectrum of **RA-34Me**

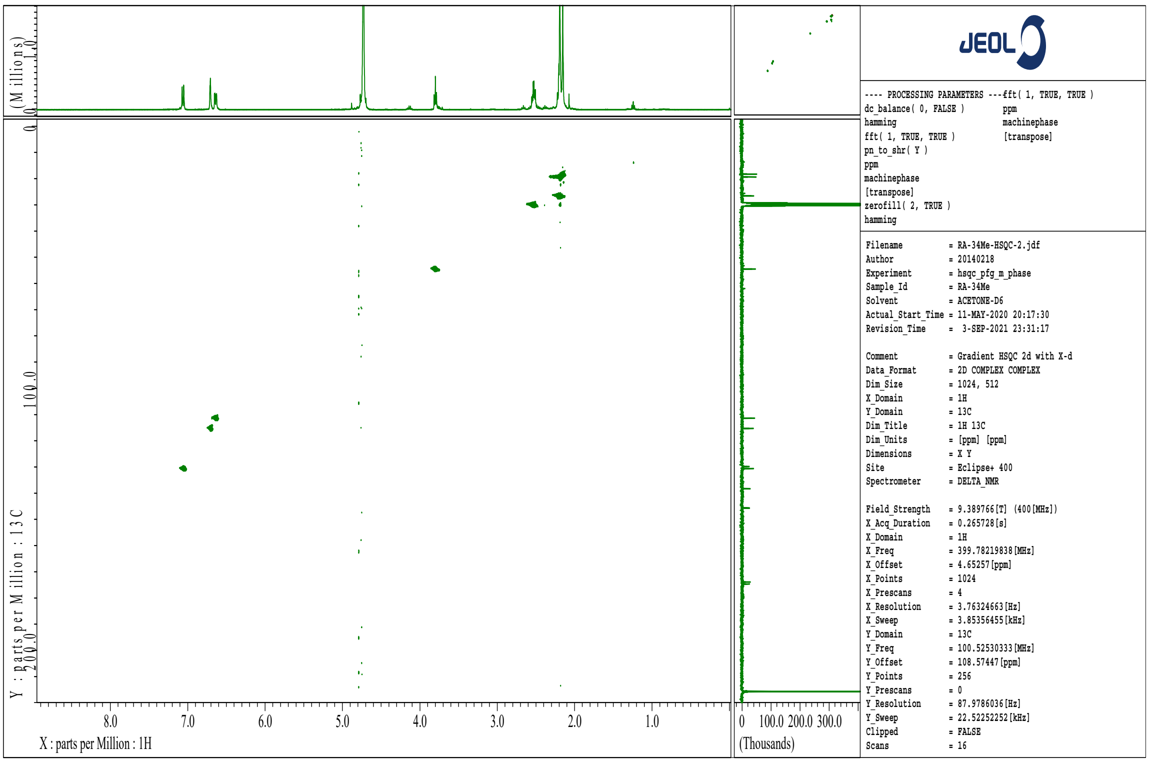

Figure S26: HSQC spectrum of **RA-34Me**

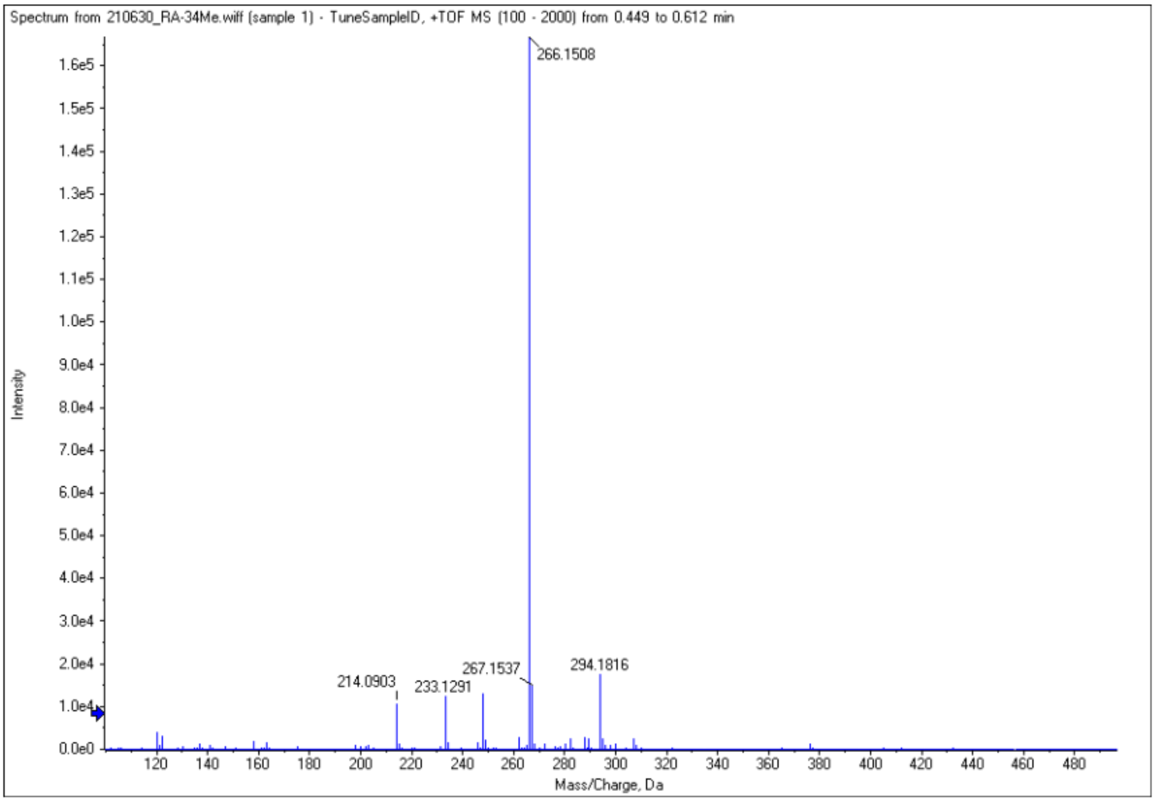

Figure S27: HRMS spectrum of **RA-34Me**

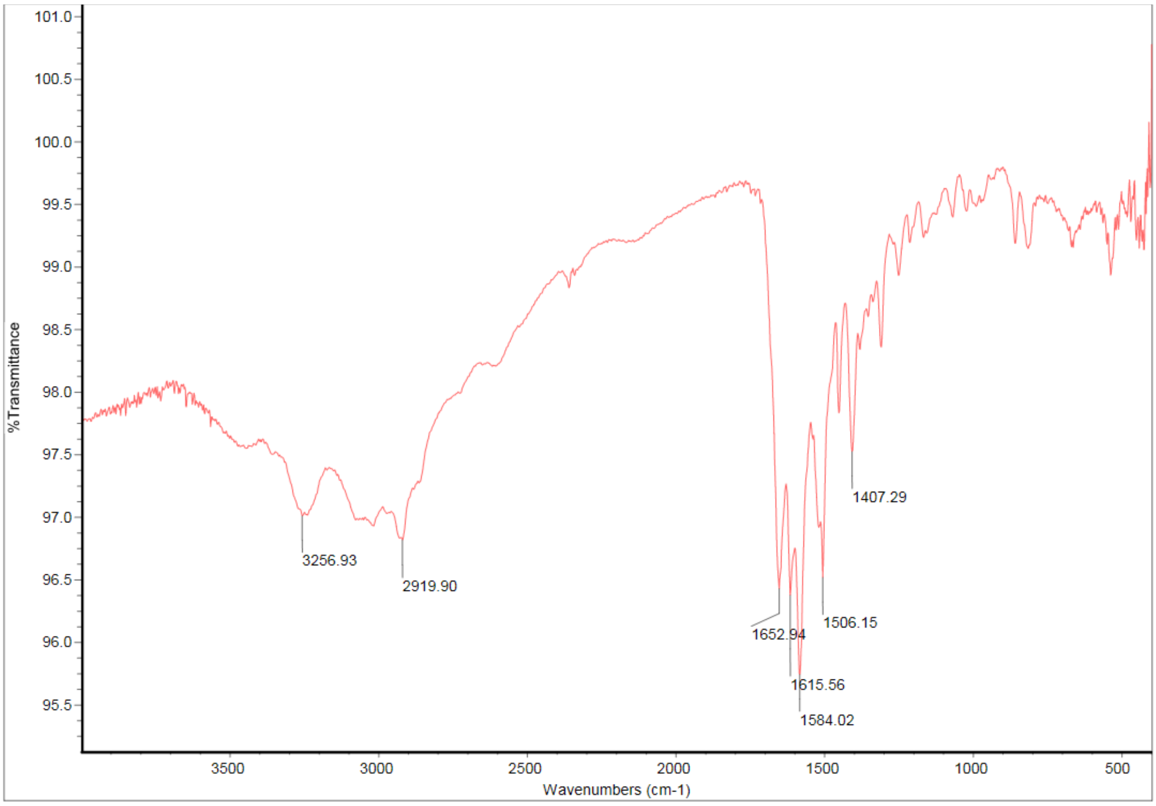

Figure S28: IR spectrum of **RA-34Me**

Position	RA-2F	
	δ_H[a] (*J* in Hz)	δ_C[b], type (*J* in Hz)
1		174.0, C
2	3.81, t (6.0)	54.5, CH
3	2.20, m	26.5, CH_2
4	2.55, m	29.9, CH_2
5		175.0, C
1'		135.3, C, d ($^2J_{CF}$ = 10.9)
2'		151.5, CF, d ($^1J_{CF}$ = 238.4)
3'	7.08-7.15, m	115.7, CH, d ($^2J_{CF}$ = 17.9)
4'	6.90-6.97, m	121.7, CH, d ($^3J_{CF}$ = 6.9)
5'	7.08-7.15, m	125.2, CH, d ($^4J_{CF}$ = 3.5)
6'	6.90-6.97, m	114.9, CH, d ($^3J_{CF}$ = 2.5)

[a]Recorded in D_2O/Acetone-*d*$_6$ (6/1), 400 MHz
[b]Recorded in D_2O/Acetone-*d*$_6$ (6/1), 100 MHz

Table S1. Assignment of NMR data of **RA-2F**

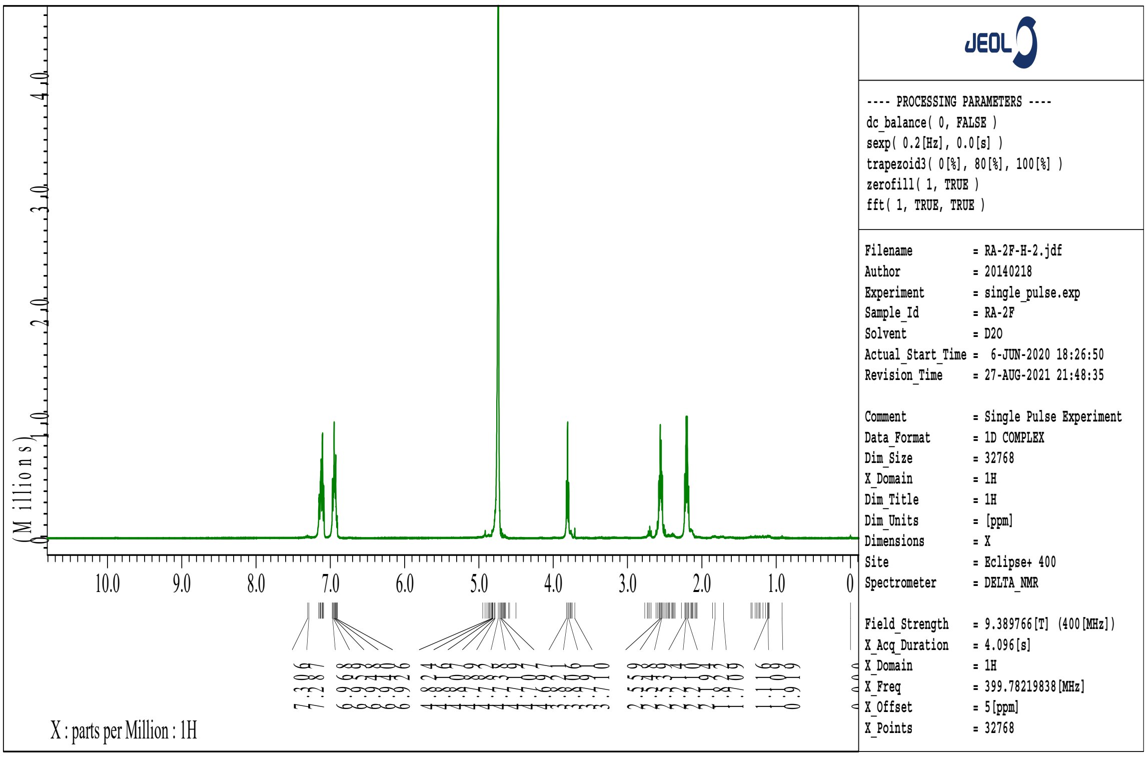

Figure S29: ^{1}H NMR (400 MHz) spectrum of **RA-2F**

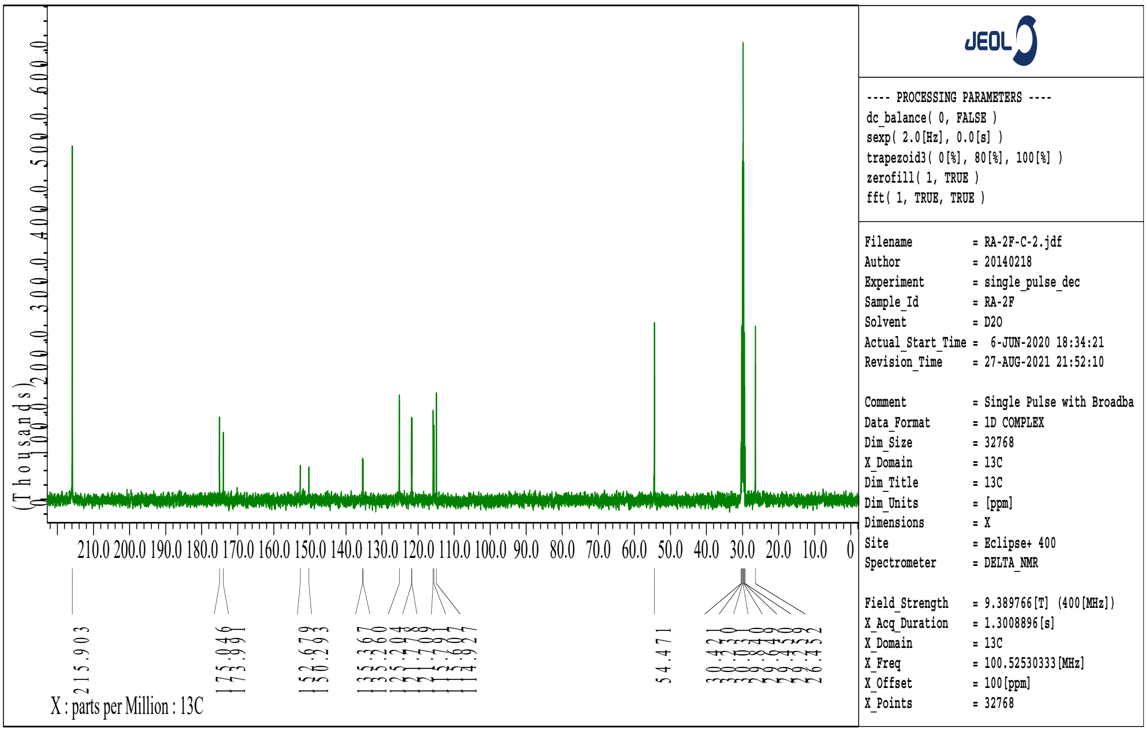

Figure S30: ^{13}C NMR (100 MHz) spectrum of **RA-2F**

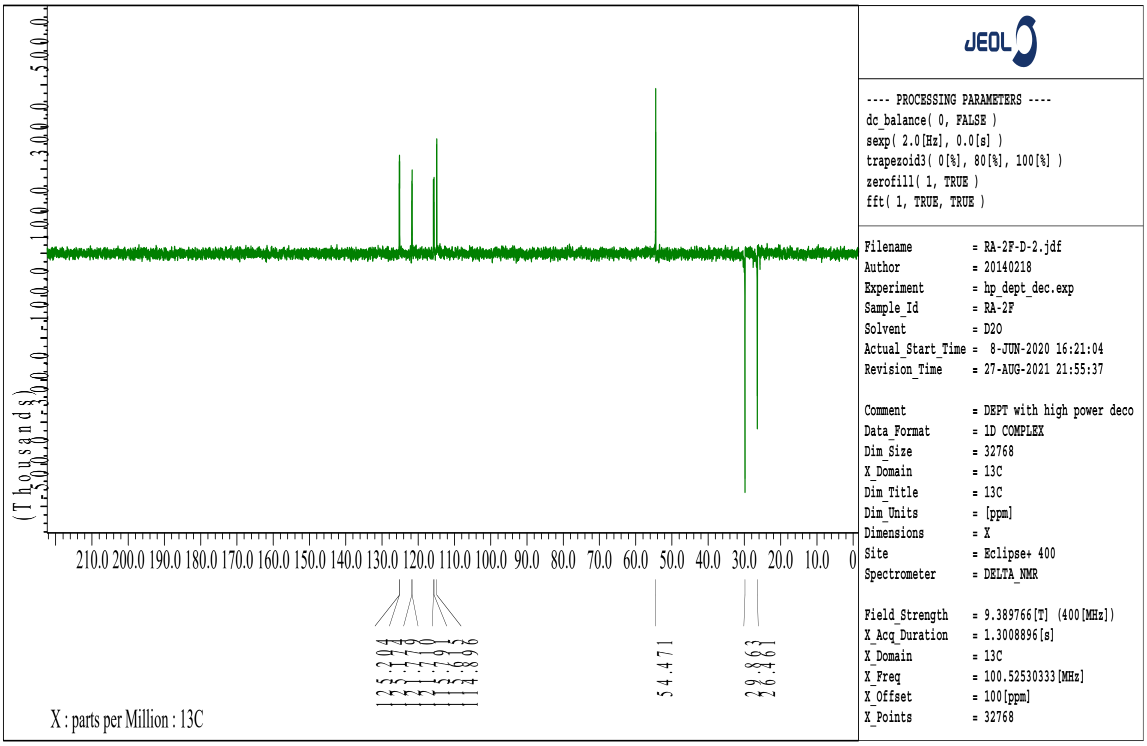

Figure S31: DEPT spectrum of **RA-2F**

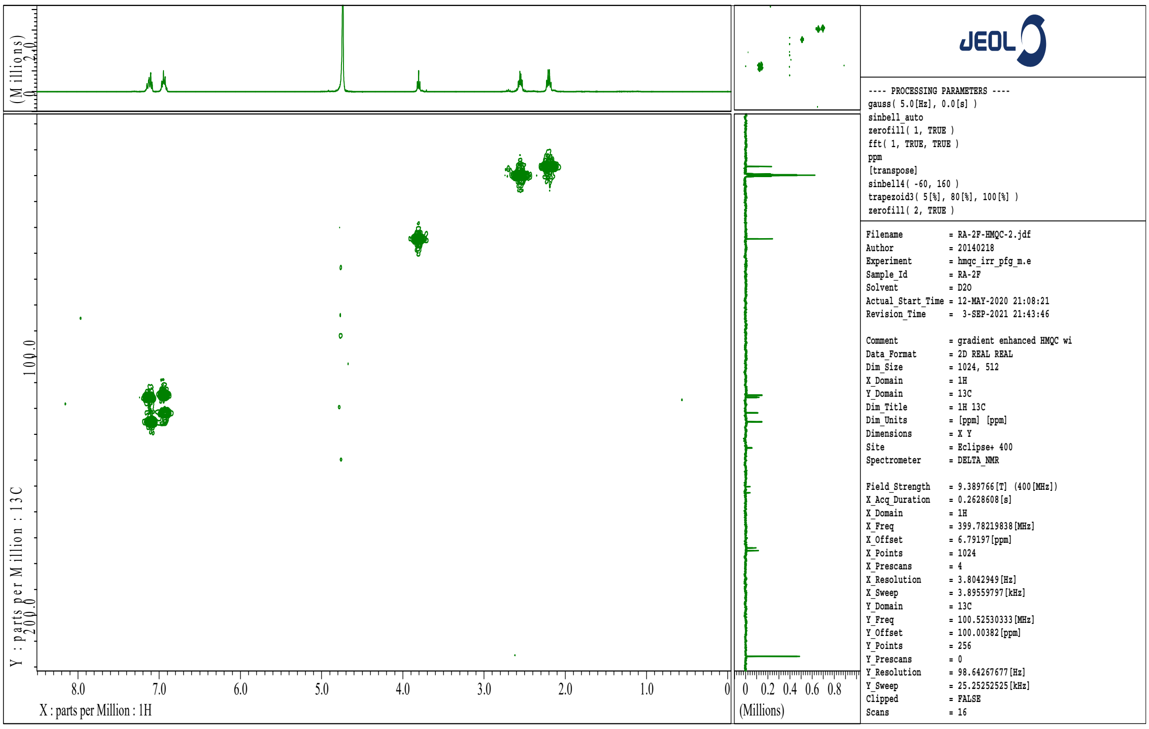

Figure S32: HMQC spectrum of **RA-2F**

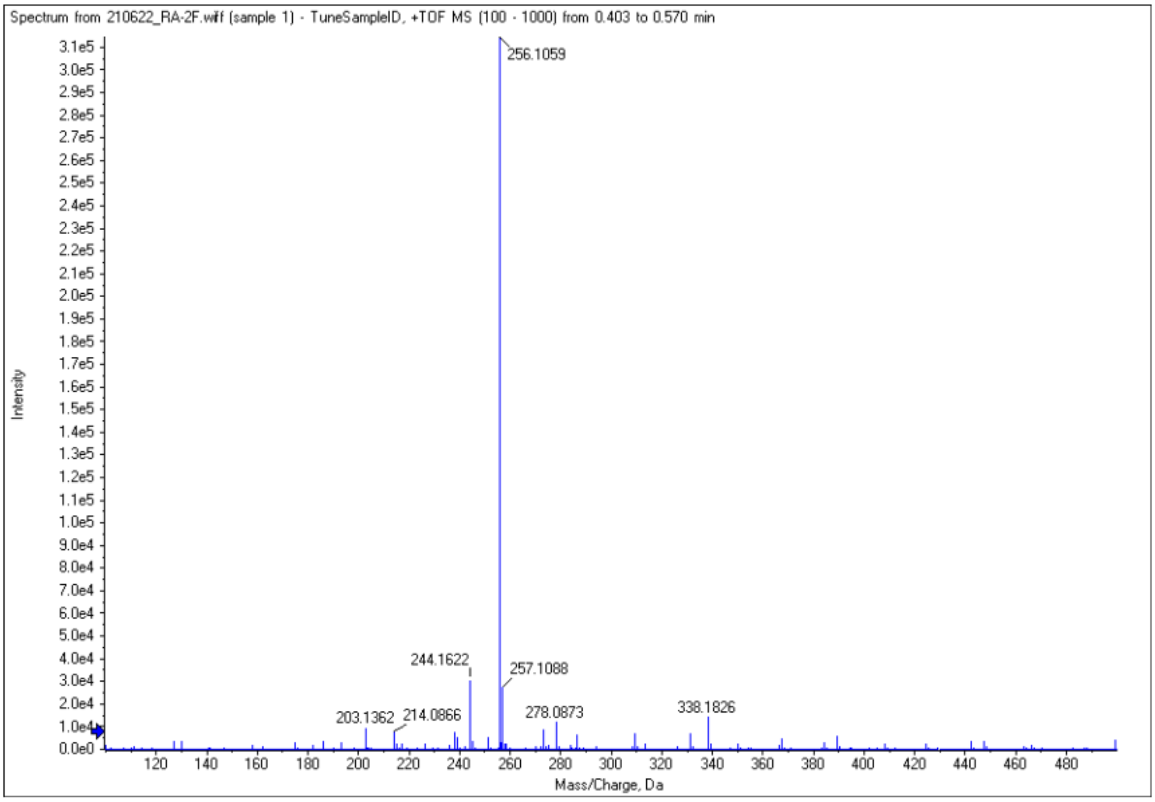

Figure S33: HRMS spectrum of **RA-2F**

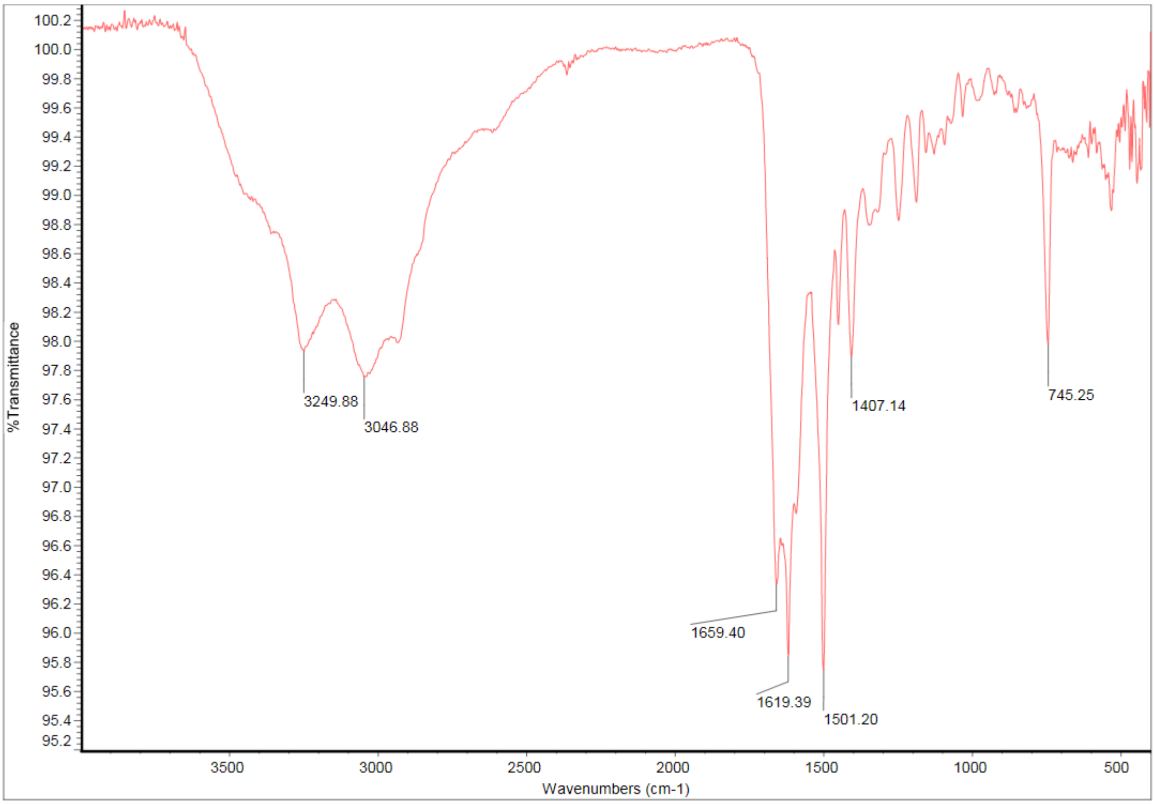

Figure S34: IR spectrum of **RA-2F**

**Position**	**RA-4F**	
	**δ_H[a] (*J* in Hz)**	**δ_C[b], type (*J* in Hz)**
**1**		**174.1, C**
**2**	**3.82, td (6.0, 1.2)**	**54.4, CH**
**3**	**2.21, m**	**26.4, CH_2**
**4**	**2.54, m**	**29.8, CH_2**
**5**		**175.0, C**
**1'**		**143.7, C, d ($^4J_{CF}$ = 2.1)**
**2'**	**6.88-6.92, m**	**115.2, CH, d ($^3J_{CF}$ = 8.0)**
**3'**	**7.04-7.09, m**	**116.1, CH, d ($^2J_{CF}$ = 22.8)**
**4'**		**158.0, C, d ($^1J_{CF}$ = 234.8)**
**5'**	**7.04-7.09, m**	**116.1, CH, d ($^2J_{CF}$ = 22.8)**
**6'**	**6.88-6.92, m**	**115.2, CH, d ($^3J_{CF}$ = 8.0)**

[a]Recorded in D_2O/Acetone-d_6 (6/1), 400 MHz
[b]Recorded in D_2O/Acetone-d_6 (6/1), 100 MHz

Table S2. Assignment of NMR data of **RA-4F**

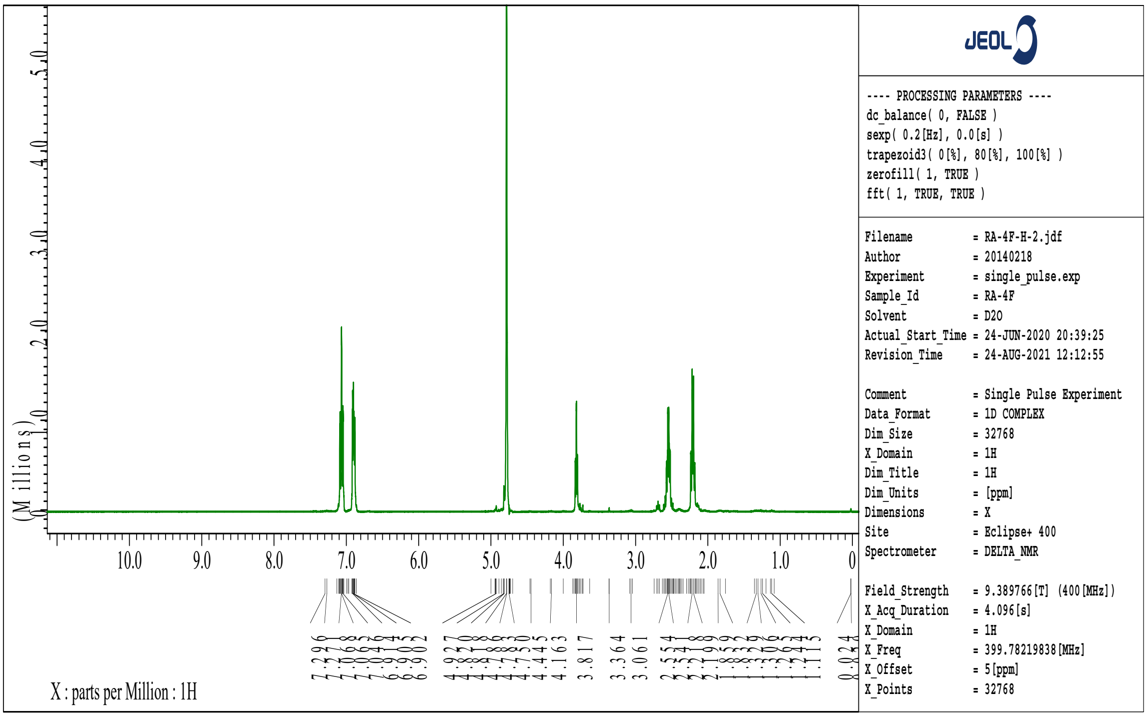

Figure S35: ^{1}H NMR (400 MHz) spectrum of **RA-4F**

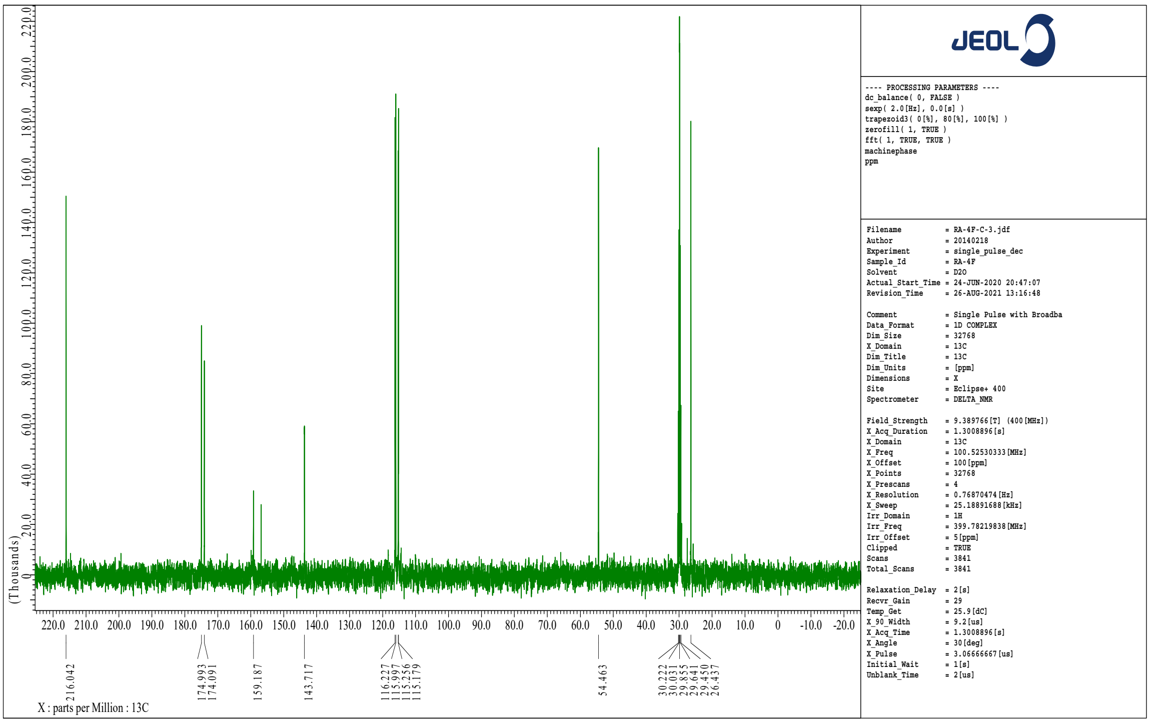

Figure S36: ^{13}C NMR (100 MHz) spectrum of **RA-4F**

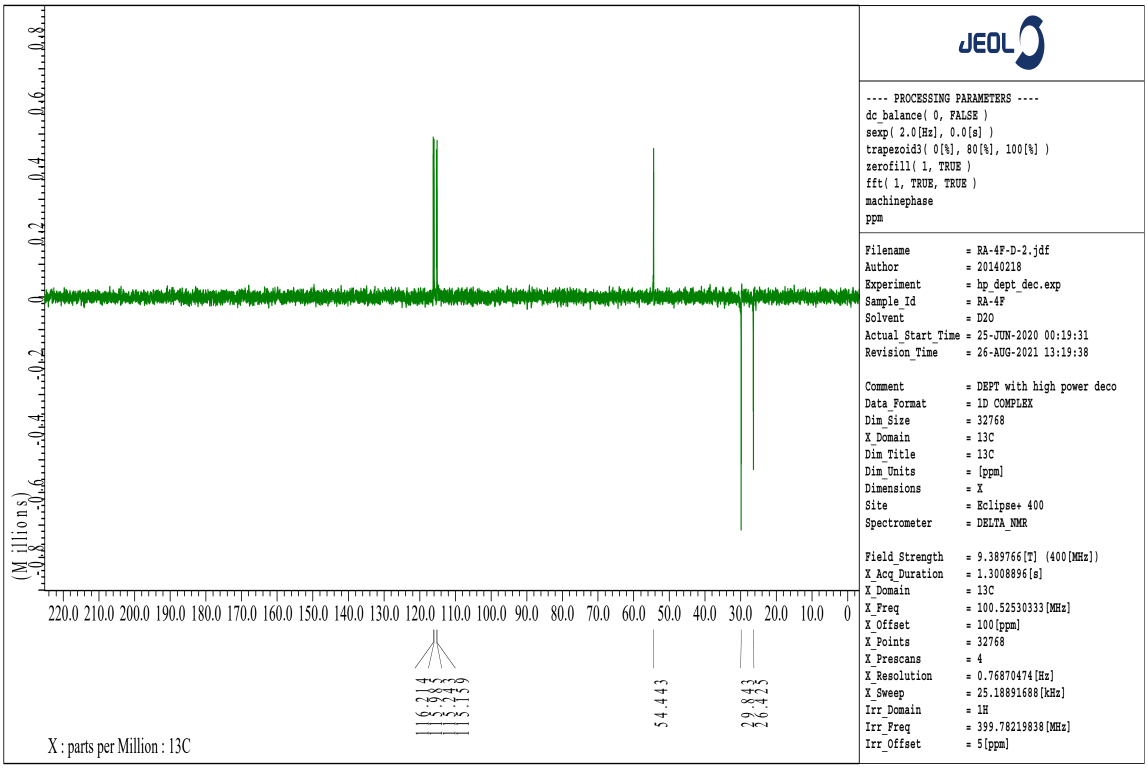

Figure S37: DEPT spectrum of **RA-4F**

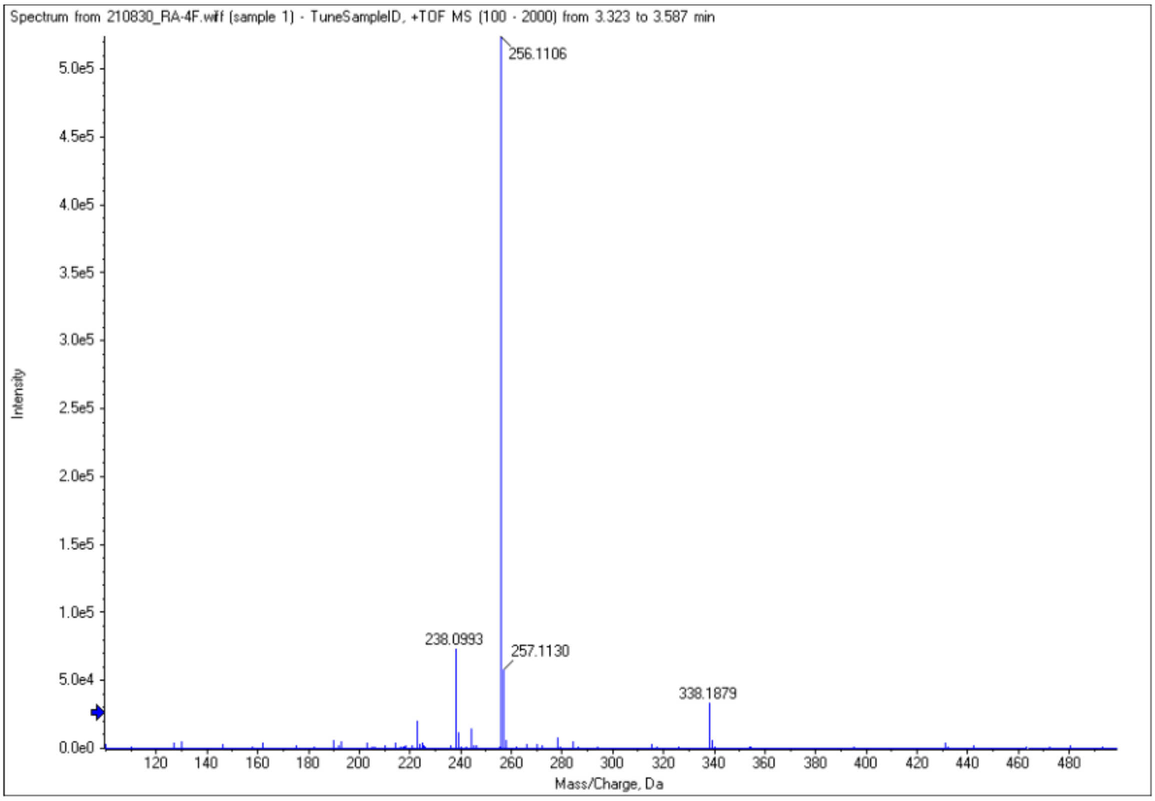

Figure S38: HRMS spectrum of **RA-4F**

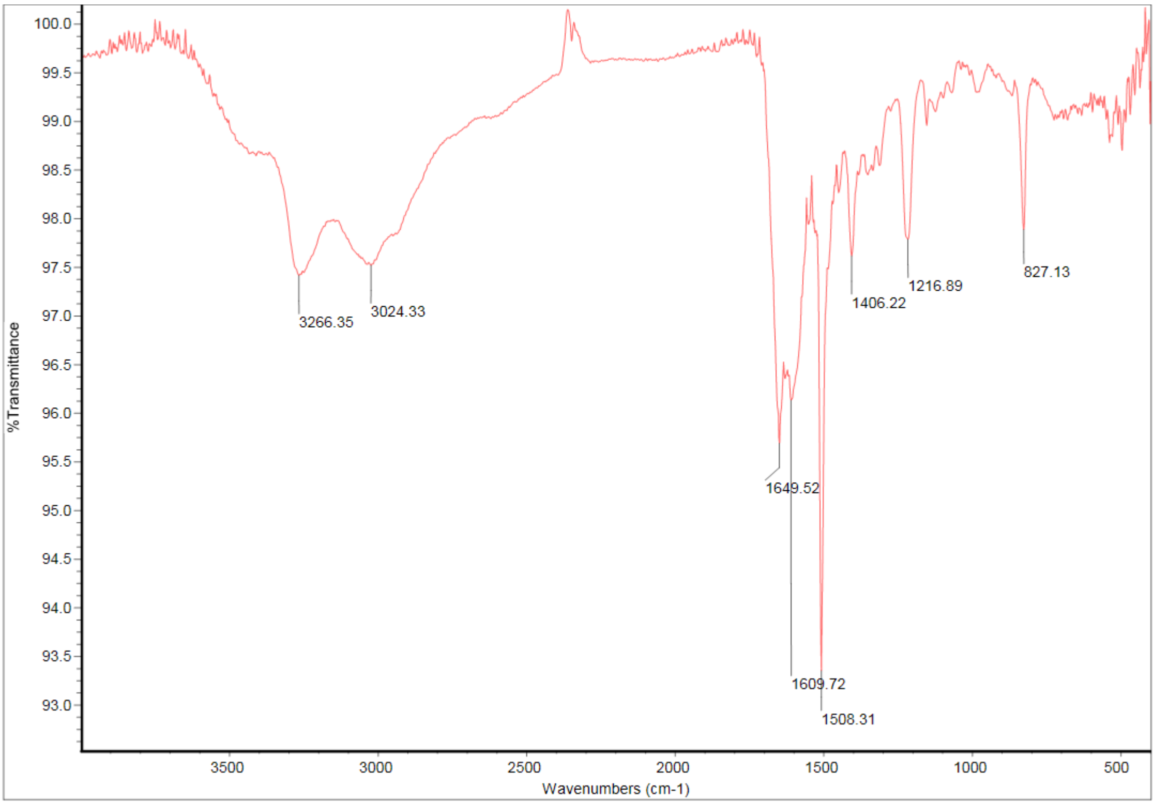

Figure S39: IR spectrum of **RA-4F**

Position	RA-24F	
	δ_H[a] (*J* in Hz)	δ_C[b], type (*J* in Hz)
1		174.1, C
2	3.79, t (6.4)	54.5, CH
3	2.18, m	26.4, CH_2
4	2.52, m	29.8, CH_2
5		175.1, C
1'		131.8, C, dd ($^4J_{CF}$ = 3.3, $^2J_{CF}$ = 11.1)
2'		157.1, CF, dd ($^3J_{CF}$ = 11.0, $^1J_{CF}$ = 237.9)
3'	6.99, m	104.3, CH, dd ($^2J_{CF}$ = 22.3, $^2J_{CF}$ = 26.9)
4'		151.2, CF, dd ($^3J_{CF}$ = 12.2, $^1J_{CF}$ = 241.9)
5'	6.89, m	111.4, CH, dd ($^4J_{CF}$ = 3.7, $^2J_{CF}$ = 22.1)
6'	6.94, m	115.8, CH, dd ($^3J_{CF}$ = 3.9, $^3J_{CF}$ = 9.4)

[a]Recorded in D_2O/Acetone-d_6 (6/1), 400 MHz
[b]Recorded in D_2O/Acetone-d_6 (6/1), 100 MHz

Table S3. Assignnment of NMR data of **RA-24F**

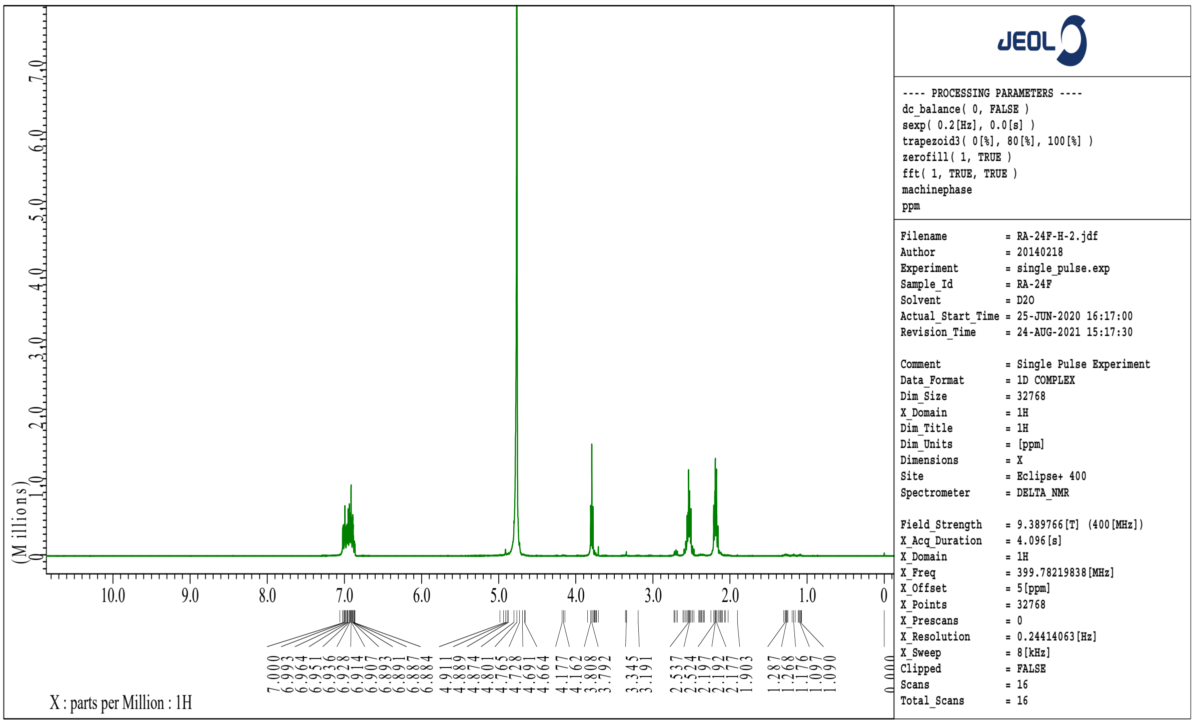

Figure S40: ^{1}H NMR (400 MHz) spectrum of **RA-24F**

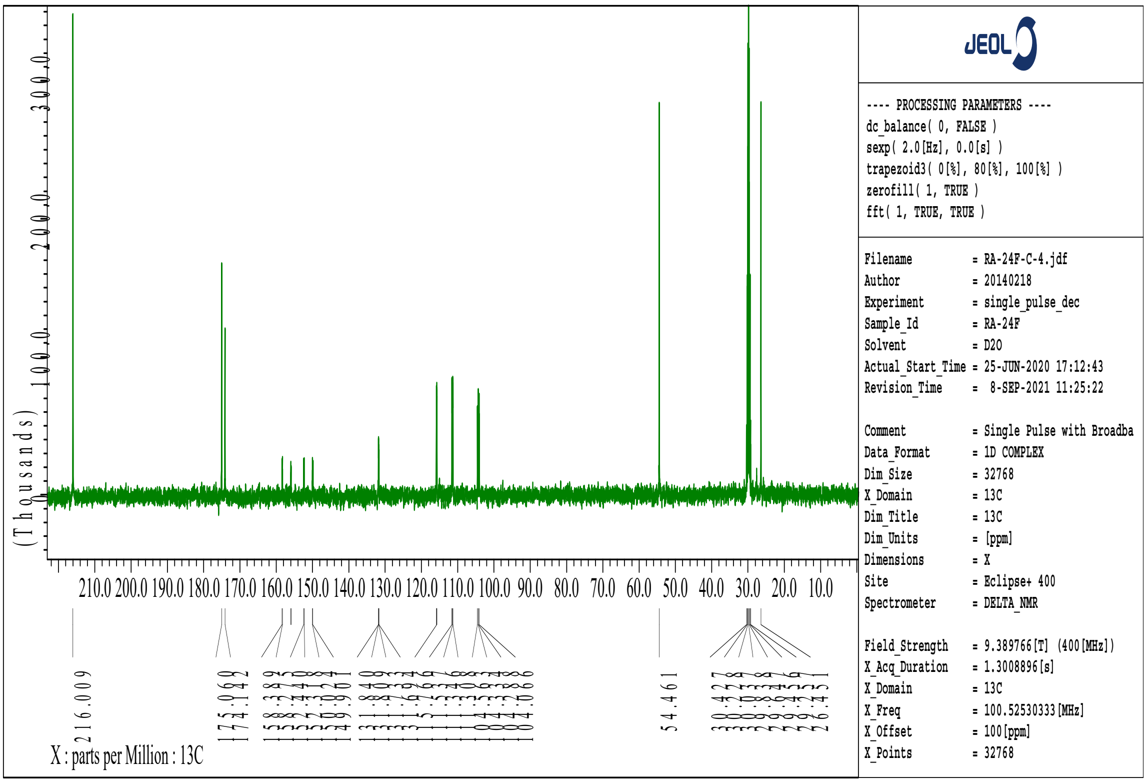

Figure S41: ^{13}C NMR (100 MHz) spectrum of **RA-24F**

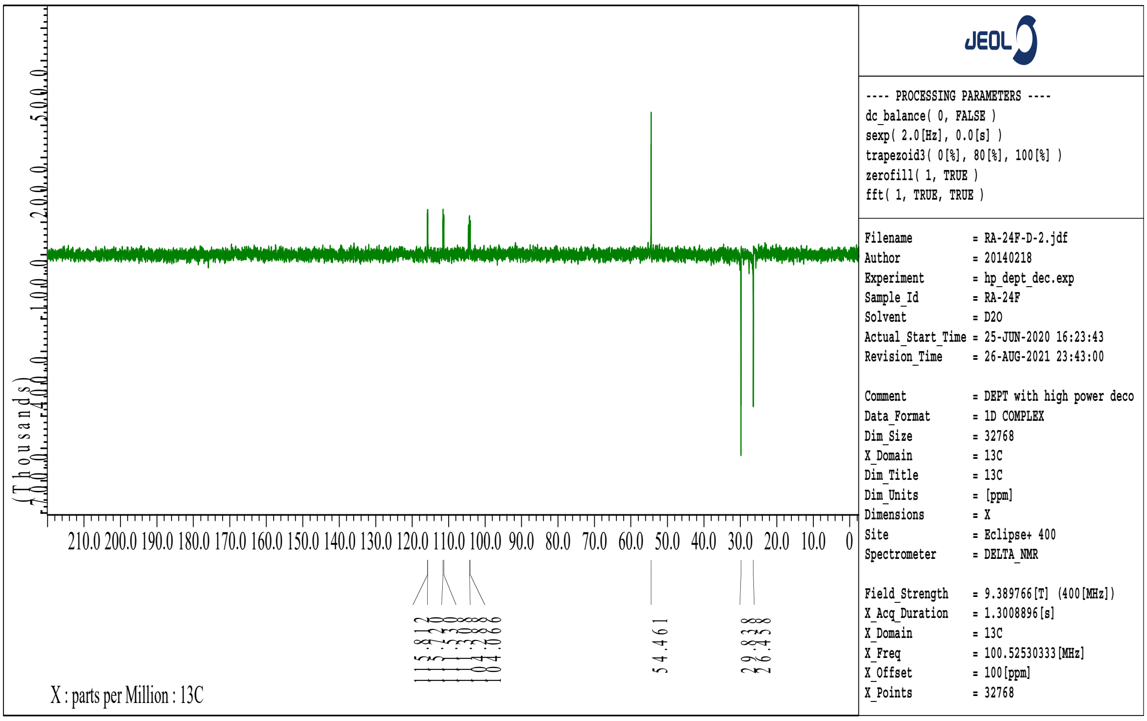

Figure S42: DEPT spectrum of **RA-24F**

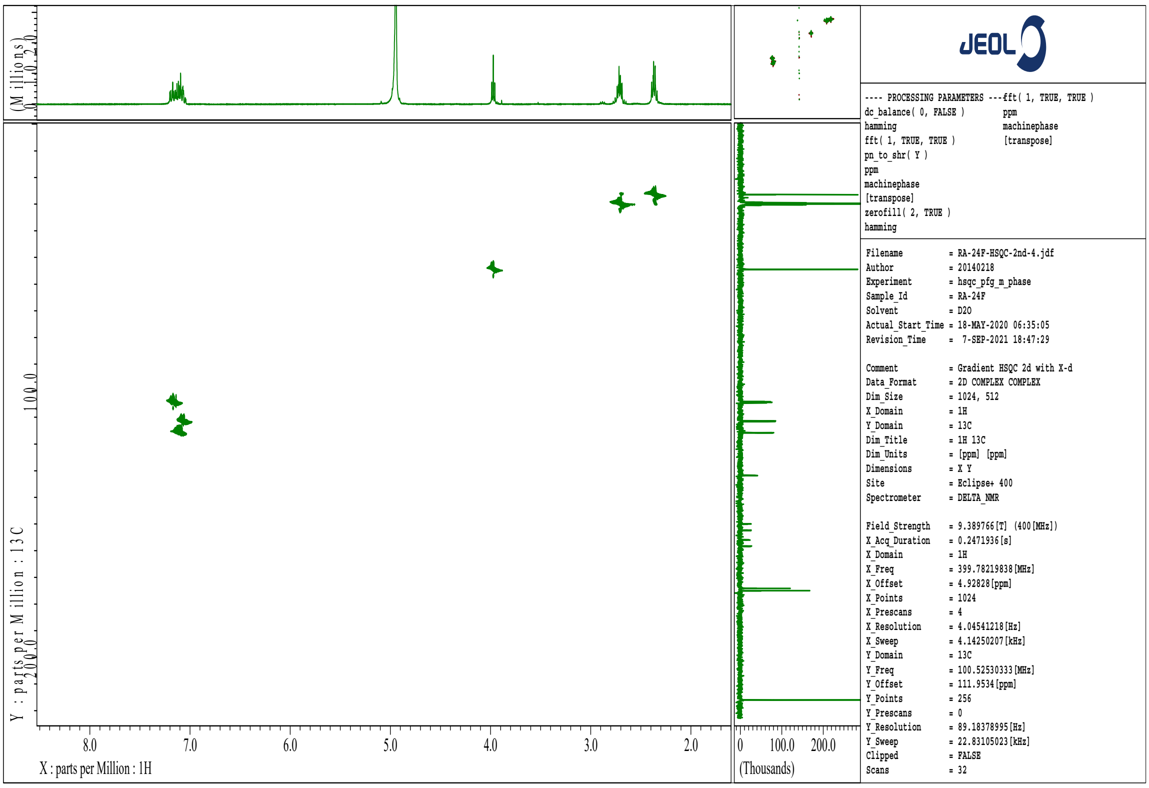

Figure S43: HSQC spectrum of **RA-24F**

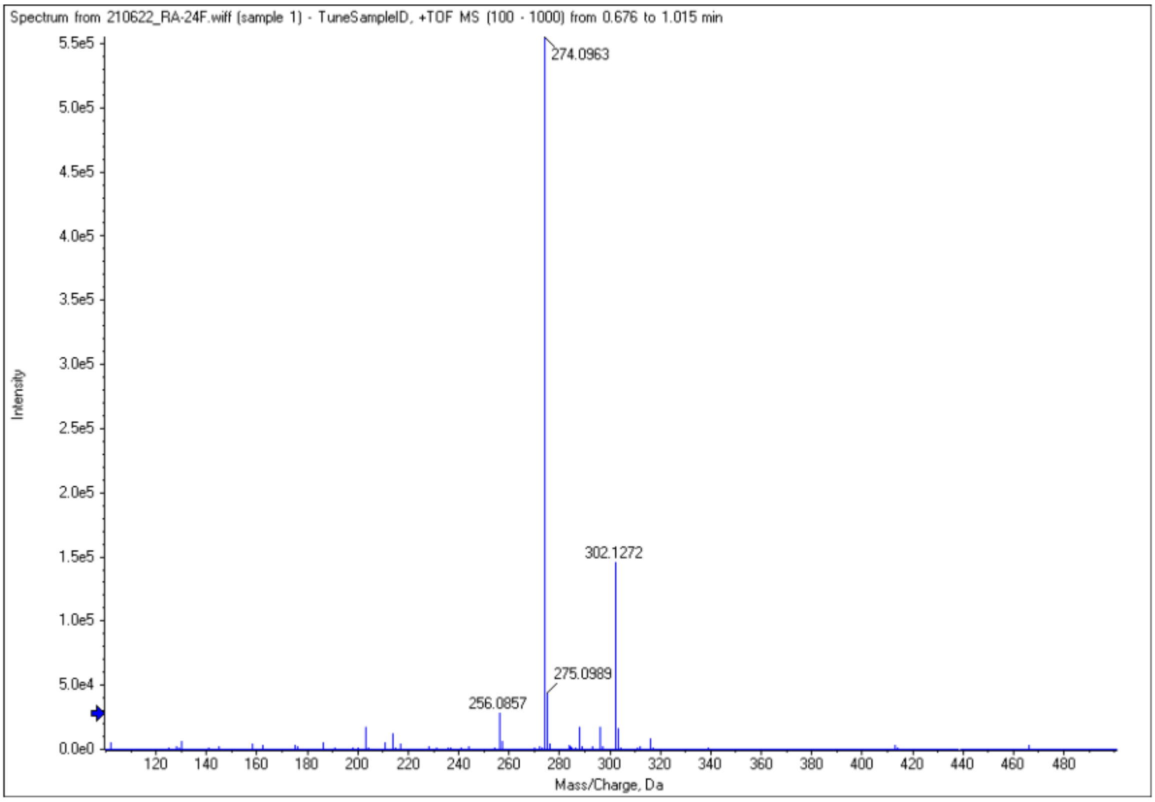

Figure S44: HRMS spectrum of **RA-24F**

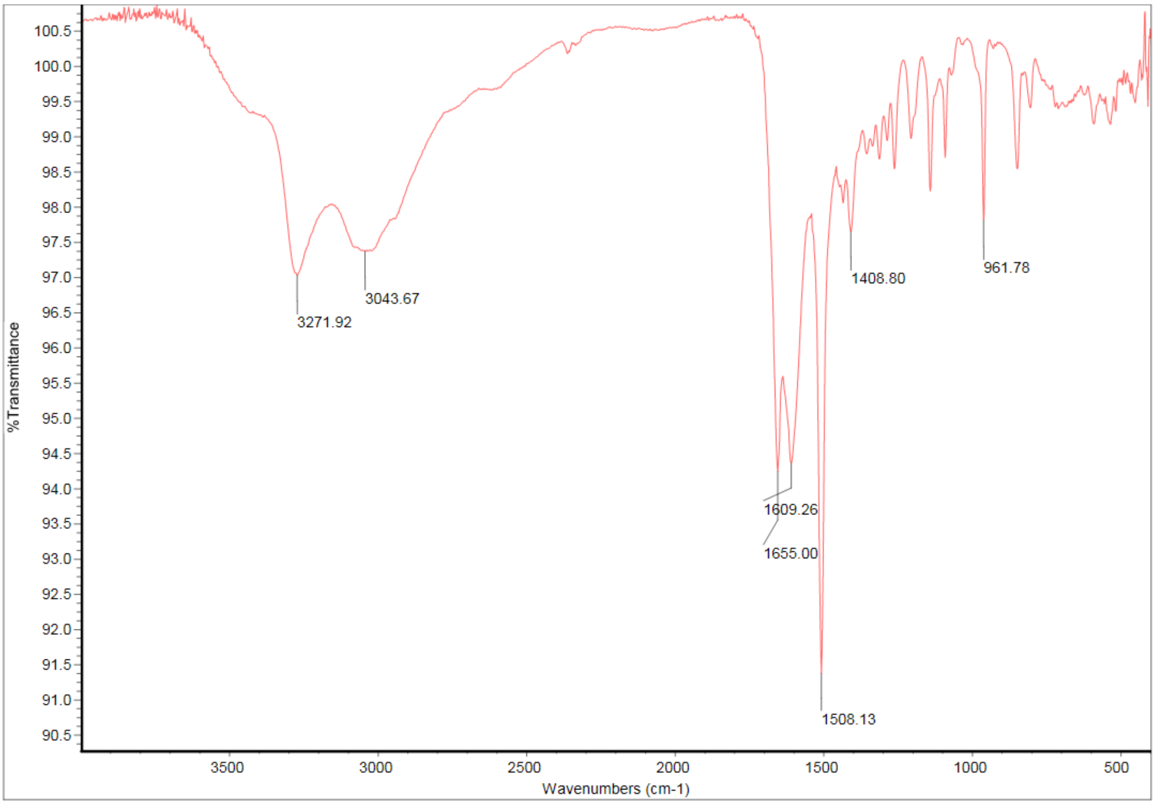

Figure S45: IR spectrum of **RA-24F**

Position	RA-PF	
	δ_H[a] (J in Hz)	δ_C[b], type (J in Hz)
1		169.5
2	3.20, t (6.4)	53.5
3	1.85, m	26.9
4	2.27, m	29.6
5		172.0
1'		124.8, C, m
2'		137.3, C, br d ($^1J_{CF}$ = 246.0)
3'		137.3, C, br d ($^1J_{CF}$ = 246.0)
4'		133.5, C, br d ($^1J_{CF}$ = 250.5)
5'		137.3, C, br d ($^1J_{CF}$ = 246.0)
6'		137.3, C, br d ($^1J_{CF}$ = 246.0)

[a]Recorded in DMSO-d6, 400 MHz
[b]Recorded in DMSO-d6, 100 MHz

Table S4. Assignment of NMR data of **RA-PF**

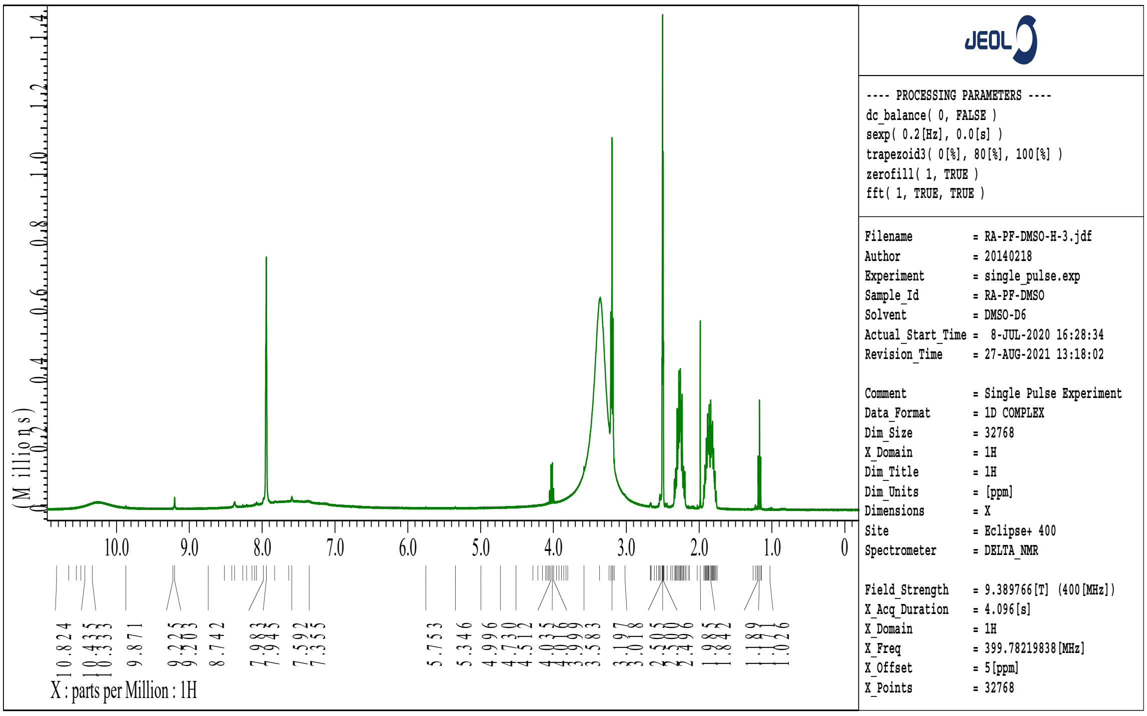

Figure S46: ^{1}H NMR (400 MHz) spectrum of **RA-PF**

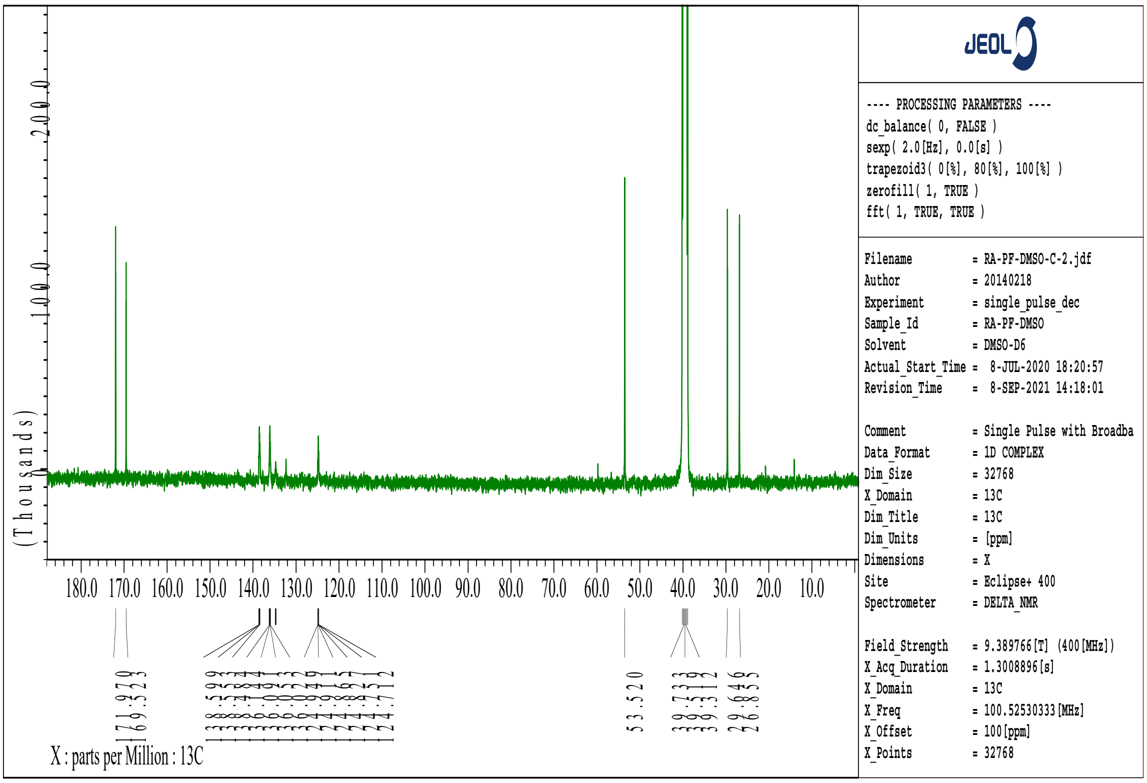

Figure S47: ^{13}C NMR (100 MHz) spectrum of **RA-PF**

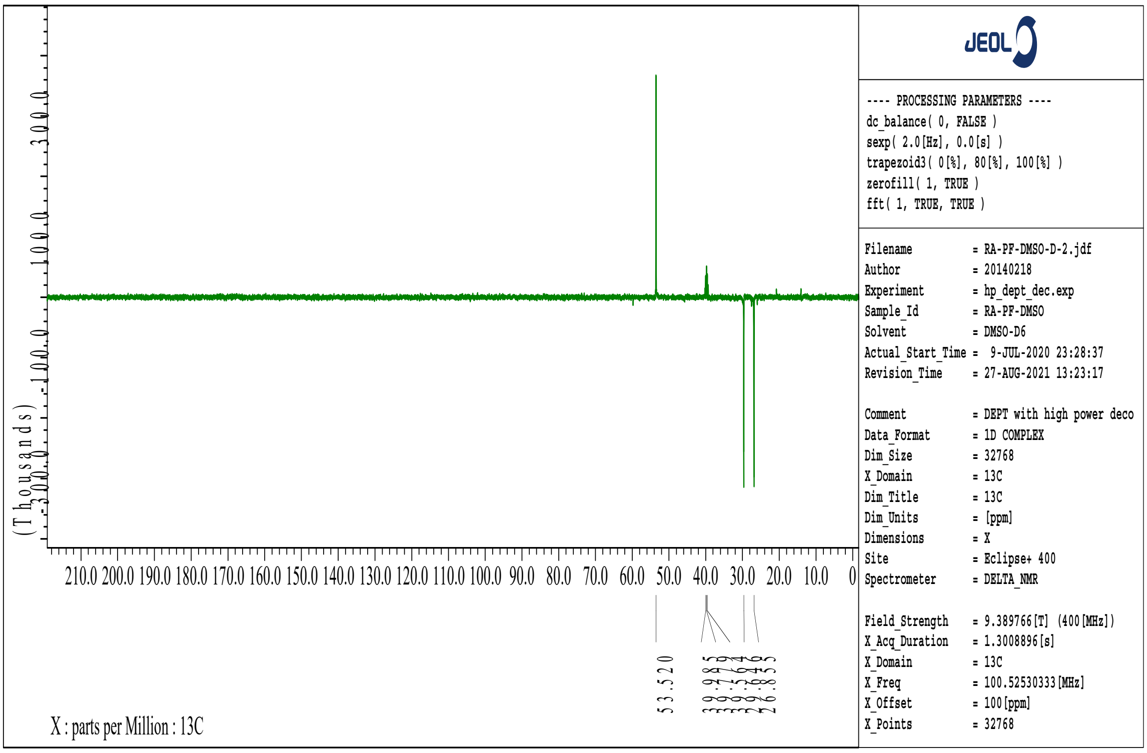

Figure S48: DEPT spectrum of **RA-PF**

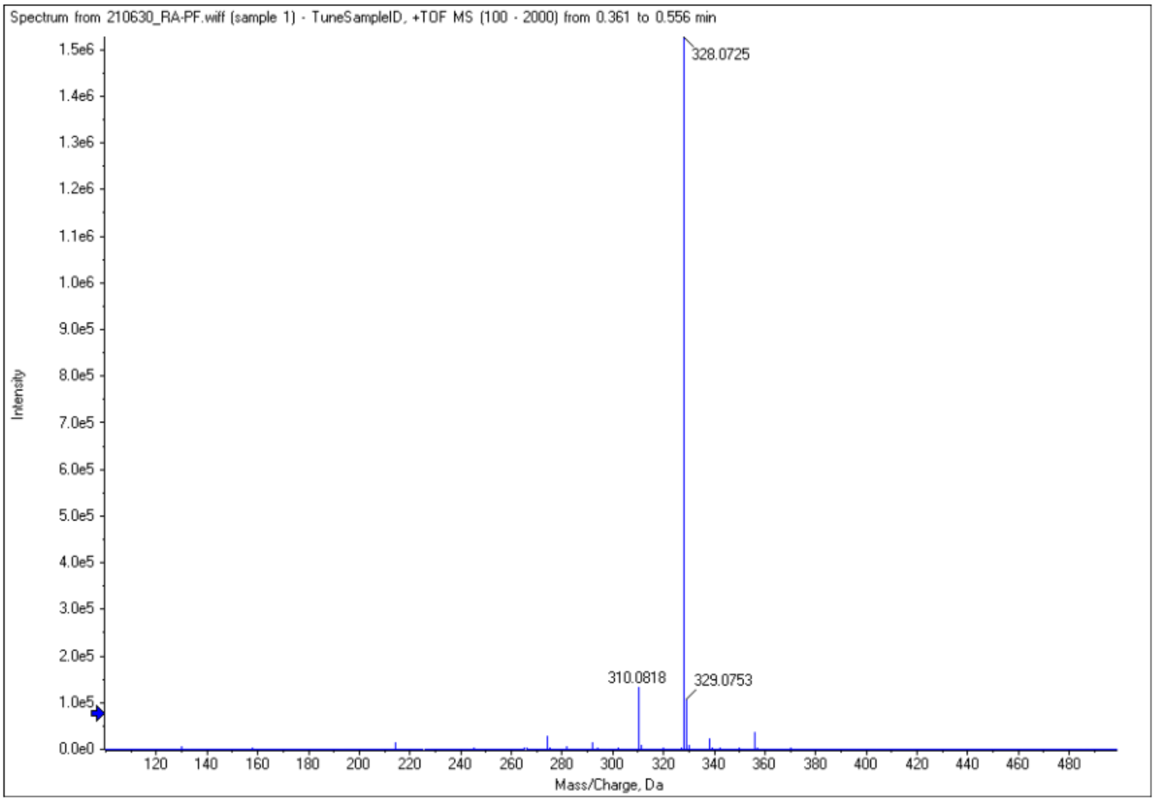

Figure S49: HRMS spectrum of **RA-PF**

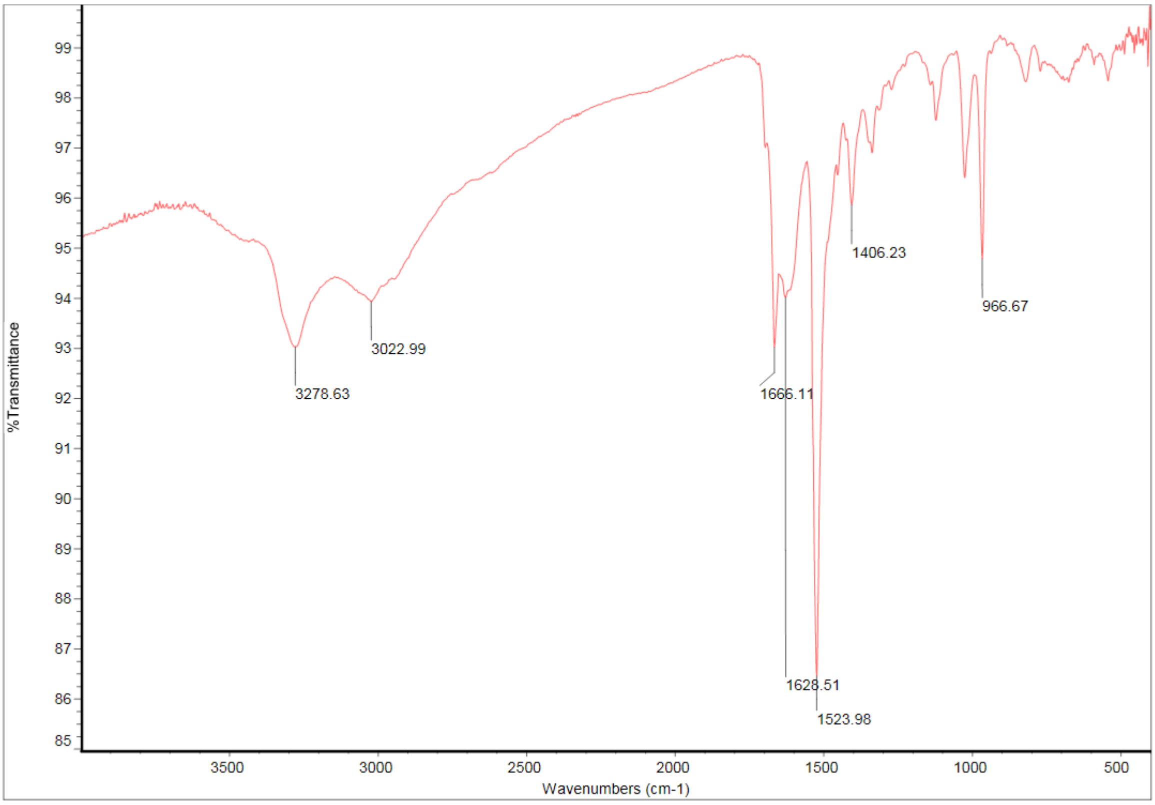

Figure S50: IR spectrum of **RA-PF**
